# Intranuclear localisation of KLF3‐AS1 under the balance of histone acetylation and methylation in BMSCs ameliorates cerebral ischaemia‒reperfusion injury by preserving microglial mitochondrial function and inhibiting inflammatory polarisation

**DOI:** 10.1002/ctm2.70870

**Published:** 2026-10-31

**Authors:** Qian Li, Xiaowei Xie, Jie Chen, Yu Cao, Dingzhou Zhou

**Affiliations:** ^1^ Department of Traditional Chinese Medicine Hunan Provincial People's Hospital (The First Affiliated Hospital of Hunan Normal University) Changsha China; ^2^ Clinical Nursing Teaching and Research Section The Second Xiangya Hospital of Central South University Changsha China; ^3^ Department of Rehabilitation Xiangya Boai Rehabilitation Hospital, Clinical Medical Technology Demonstration Base for Cerebrovascular Disease Rehabilitation in Hunan Province Changsha China; ^4^ Department of Comprehensive Surgery Hunan Provincial People's Hospital (The First Affiliated Hospital of Hunan Normal University) Changsha China; ^5^ Department of Neurosurgery Hunan Provincial People's Hospital (The First Affiliated Hospital of Hunan Normal University) Changsha China

**Keywords:** bone marrow‐derived mesenchymal stem cells, cerebral ischaemia‒reperfusion injury, KLF3‐AS1, microglial polarisation, SIRT3

## Abstract

**Background:**

Ischaemic stroke occurs when blood flow to the brain is obstructed. Rapid restoration of blood supply frequently induces cerebral ischaemia‒reperfusion injury (CIRI). Bone marrow mesenchymal stem cell (BMSC)‐derived exosomes exhibit promising therapeutic potential for CIRI. In‐depth exploration of their therapeutic mechanisms holds crucial significance for CIRI management.

**Methods:**

The middle cerebral artery occlusion/reperfusion (MCAO) model was established in mice. An in vitro ischaemic stroke model was established by treating BV‐2 cells with oxygen‒glucose deprivation/reoxygenation (OGD/R). Exosomes were isolated from BMSCs and intranasally administrated into mice or added into BV‐2 cells. Neurological deficits, grasping and coordination function were evaluated. Cell viability, apoptosis, proliferation and migration were examined. The localisation of KLF3‐AS1 was examined by combined staining of immunofluorescence and fluorescence in situ hybridisation. Intracellular Ca^2+^ and adenosine triphosphate levels, mitochondrial reactive oxygen species and membrane potential were assessed.

**Results:**

KLF3‐AS1 enhanced BMSC viability, proliferation and migration, accompanied by epigenetic reprogramming at its promoter characterised by increased H3K27 acetylation (H3K27Ac) and decreased H3K27 trimethylation (H3K27Me3). BMSC‐derived KLF3‐AS1‐enriched exosomes indicated therapeutic efficacy in both in vivo and in vitro models of cerebral ischaemia. In MCAO‐induced mice, these exosomes alleviated neurological deficits and shifted microglial polarisation from pro‐inflammatory M1‐like to anti‐inflammatory M2‐like phenotypes. Similar effects were observed in OGD/R‐treated BV‐2 microglia, where KLF3‐AS1‐enriched exosomes attenuated oxidative stress and mitochondrial dysfunction while upregulating Silent Information Regulator 3 (SIRT3) expression. Mechanistically, KLF3‐AS1 facilitated ETS Variant Transcription Factor 4 (ETV4) nuclear translocation to activate SIRT3 transcription in microglia, and inhibition of SIRT3 abolished the neuroprotective effects of exosomal KLF3‐AS1.

**Conclusion:**

BMSC‐derived exosomes delivering KLF3‐AS1 ameliorates CIRI by preserving microglial mitochondrial function and inhibiting inflammatory polarisation, highlighting KLF3‐AS1 as a critical epigenetic regulator and exosome‐mediated therapeutic agent for CIRI through the ETV4/SIRT3 axis.

**Key points/Highlights:**

1. KLF3‐AS1 enhances bone marrow mesenchymal stem cell (BMSC) function via promoter H3K27Ac/H3K27Me3 reprogramming.
2. KLF3‑AS1 directly binds ETS Variant Transcription Factor 4 (ETV4) to activate Silent Information Regulator 3 (SIRT3), preserving mitochondrial integrity.
3. BMSC‑derived exosomal KLF3‑AS1 promotes M2 microglial polarisation and ameliorates cerebral ischaemia–reperfusion injury.

## Graphical abstract

Epigenetic activation of KLF3‐AS1 in BMSCs, characterised by increased H3K27 acetylation and decreased H3K27 trimethylation at its promoter, enhances BMSC viability, proliferation and migration. Exosomal KLF3‐AS1 is delivered to microglia, which mechanistically promotes ETV4 nuclear translocation to activate SIRT3 transcription, preserving microglial mitochondrial function and suppressing inflammatory polarisation, ultimately alleviating CIRI. This identifies the KLF3‐AS1/ETV4/SIRT3 axis as a key epigenetic and therapeutic pathway in CIRI.

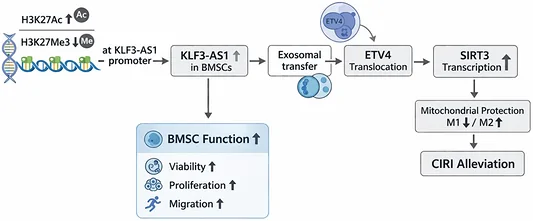

## 1 INTRODUCTION

Ischaemic stroke, accounting for approximately 85% of all strokes, occurs due to the sudden occlusion of cerebral blood flow, typically caused by thromboembolism or thrombosis within cerebral arteries, and is a leading cause of death and disability globally.^1^, ^2^ The primary treatment of ischaemic stroke focuses on rapid restoration of cerebral blood flow to salvage the ischaemic penumbra and prevent secondary neuronal injury.^3^ Despite the restoration of blood flow to ischaemic brain regions is essential to restore oxygen supply, it also causes severe neurological damage known as cerebral ischaemia‒reperfusion injury (CIRI) through various mechanisms such as calcium overload, oxidative stress, blood‒brain barrier disruption, apoptosis and ferroptosis.^4^, ^5^ CIRI causes a poor prognosis for patients with ischaemic stroke and imposes a critical challenge for stoke treatment. However, the complex pathogenesis of CIRI has hindered the development of effective therapeutic approaches. Therefore, a better understanding of the mechanisms of CIRI contributes to developing effective therapeutics for the management of ischaemic stroke and subsequent CIRI.

Bone marrow mesenchymal stem cell (BMSC)‐derived exosomes exhibit promising therapeutic potential for CIRI. BMSC‐derived exosomes shift microglial polarisation towards the anti‐inflammatory M2 phenotype, thus attenuating CIRI‐induced pyroptosis and neuroinflammation.^6^ These nano‐sized vesicles derived from BMSCs, enriched with bioactive molecules such as lipids, proteins and nuclear acids, exert protective effects on CIRI. BMSC‐derived exosomes delivering miR‐145 attenuate CIRI by downregulating Forkhead Box O1 (FOXO1).^7^ Nuclear factor erythroid 2‐related factor 2 (NRF2)‐enriched exosomes derived from BMSCs alleviate CIRI via activating Lin‐28 Homologue A (Lin28a) expression.^2^ Ubiquitin Specific Peptidase 10 (USP10)‐enriched BMSC‐derived exosomes protect against CIRI by stabilising Solute Carrier Family 7 Member 11 (SLC7A11).^8^ Thus, BMSC‐derived exosomes have gained much attention as a promising cell‐free approach for CIRI. A previous study has suggested that BMSC‐derived exosomes loaded with tetramethylpyrazine significantly improve cerebral ischaemic damage by repressing oxidative stress and neuronal apoptosis.^9^ These findings highlight BMSC‐derived exosomes as promising candidates for CIRI management.

Growing evidence indicates that long non‐coding RNAs (lncRNAs) are critical cargo components within BMSC‐derived exosomes.^10^, ^11^, ^12^ BMSC‐derived exosomal lncRNA Krüppel‐like factor 3 antisense RNA 1 (KLF3‐AS1) alleviates endothelial dysfunction and neurological deficits following subarachnoid haemorrhage.^13^ Importantly, it has been reported that exosomal KLF3‐AS1 derived from BMSCs protects against CIRI by facilitating autophagy through the ETS Variant Transcription Factor 4 (ETV4)/Silent Information Regulator 1 (SIRT1) axis.^14^ Additionally, BMSC‐derived exosomes delivering KLF3‐AS1 ameliorate CIRI‐induced inflammatory damage via the miR‐206/USP22 axis by stabilising SIRT1.^15^ These findings suggest that upregulating KLF3‐AS1 expression in BMSC‐derived exosomes represents a potential therapeutic strategy for CIRI. The dysregulated expression of lncRNAs results from transcriptional activation that is modulated by histone acetylation and methylation modifications.^10^ The presence of histone acetylation and methylation modifications at the KLF3‐AS1 promoter in BMSCs has not yet been elucidated.

Microglia, the primary innate immune cells in the brain, undergo polarisation driven by microenvironmental changes during CIRI, and activation of pro‐inflammatory M1 microglia exacerbates CIRI.^16^ Accumulating evidence suggests that shifting microglial polarisation from pro‐inflammatory M1 to anti‐inflammatory M2 phenotypes ameliorates CIRI,^17^, ^18^ positioning this process as a promising therapeutic target. A previous single study has reported that BMSC‐derived exosomes promote M1‐to‐M2 microglial repolarisation to attenuate CIRI,^6^ with the underlying regulatory mechanisms remaining unexplored. These studies underscore the critical need to investigate whether exosomal KLF3‐AS1 regulates microglial polarisation in CIRI, which will provide essential experimental evidence for translating BMSC‐derived exosome‐based therapies into clinical practice. Although prior studies have demonstrated that BMSC‐derived exosomal KLF3‐AS1 exerts neuroprotective effects against CIRI through two distinct SIRT1‐related mechanisms, whether KLF3‐AS1 regulates microglial mitochondrial function and inflammatory polarisation through SIRT3, a NAD^+^‐dependent deacetylase primarily localised in mitochondria and critically involved in metabolic regulation, remains unknown. Moreover, the epigenetic regulation of KLF3‐AS1 expression in BMSCs has not been explored. ETV4, a member of the ETS transcription factor family, has been implicated in various cellular processes. Recent studies have demonstrated that ETV4 protects against CIRI by suppressing neuronal ferroptosis through transcriptional upregulation of YBX1, and its stability is regulated by METTL3‐mediated m6A modification in microglia during neuroinflammation.^19^, ^20^ However, whether ETV4 mediates KLF3‐AS1‐induced microglial mitochondrial regulation and inflammatory polarisation remains unexplored. In this study, we aimed to investigate (1) whether histone acetylation and methylation modifications at the KLF3‐AS1 promoter regulate its expression in BMSCs, and (2) whether exosomal KLF3‐AS1 protects against CIRI by preserving microglial mitochondrial function and modulating inflammatory polarisation through the ETV4/SIRT3 axis.

Here, we found that the histone acetylation and demethylation at the KLF3‐AS1 promoter synergistically upregulate KLF3‐AS1 expression in BMSC‐derived exosomes, thereby enhancing BMSC migration and functionality. Intriguingly, KLF3‐AS1‐enriched exosome derived from BMSCs ameliorates mitochondrial dysfunction and promotes microglial polarisation towards the anti‐inflammatory M2 phenotype by facilitating ETV4 nuclear translocation and activating SIRT3 expression, ultimately exerting protective effects against CIRI. Our study not only reveals a novel mechanism by which exosomal KLF3‐AS1 derived from BMSCs ameliorates CIRI but also provides critical theoretical support for addressing current clinical challenges in CIRI management and novel therapeutic targets and strategies for CIRI.

## 2 METHODS

### 2.1 BMSC isolation, culture and characterisation

Human BMSCs (ATCC® PCS‐500‐012) were cultured in α‐MEM supplemented with 10% fetal bovine serum and 1% penicillin–streptomycin. Cells were maintained at 37°C in a humidified incubator with 5% CO_2_. Primary murine BMSCs were isolated from tibiae and femora of 8‐week‐old male C57BL/6 mice as previously described.^15^ Briefly, mice were anaesthetised, and bone marrow cells were flushed and centrifuged at 1000 rpm for 5 min to pellet cells. Cells were then cultured in Dulbecco's Modified Eagle Medium (DMEM)/10% fetal bovine serum (FBS, Thermo Fisher Scientific) under standard conditions (37°C, 5% CO_2_). At passage 3, cells were characterised via flow cytometry using species‐appropriate lineage‐specific markers: positive for CD105 and CD90 and negative for CD34, CD45 and HLA‐DR (murine BMSCs lack HLA‐DR expression). Human BMSCs from passages 3–8 were utilised for subsequent experiments to explore the biological functions (involving in cell viabillity, proliferation and migration) and upstream molecular mechanisms of KLF3‑AS1. For KLF3‐AS1 or KDM4A overexpression, BMSCs were transfected with the pcDNA3.1‐KLF3‐AS1 or pcDNA3.1‐KDM4A using Lipofectamine 3000 reagent (Thermo Fisher Scientific). For KAT6A knockdown, BMSCs were transfected with the short hairpin RNA against KAT6A (sh‐KAT6A, RiboBio) using Lipofectamine 3000 reagent. For C646 treatment, BMSCs were treated with C646 (Selleck) at 20 µM for 24 h. Considering interspecies differences, murine BMSCs were applied for exosome isolation and characterization, in vivo exosome administration, and in vitro treatment of BV‐2 microglia.

### 2.2 Exosome isolation and characterisation

BMSCs were maintained in exosome‐depleted FBS (Thermo Fisher Scientific)‐supplemented complete medium, and exosomes were isolated from the BMSC culture medium using Total Exosome Isolation Reagent (Thermo Fisher Scientific). Exosome morphology was examined by transmission electron microscopy (TEM). Exosomal markers CD63, CD81, TSG101 and the negative marker Calnexin were detected by Western blotting. Particle size distribution was analysed by nanoparticle tracking analysis (NTA). Exosomes were labelled with PKH26 (MCE) or DiI (Thermo Fisher Scientific) at 1 µM for 15 min, washed and added into cells to examine exosome internalisation.

### 2.3 BV‐2 cell culture, transfection and treatment

BV‐2 microglia were provided by Cytion and cultured in DMEM/10% FBS (Thermo Fisher Scientific) under standard conditions (37°C, 5% CO_2_). For KLF3‐AS1 overexpression and ETV4 and SIRT3 knockdown, BV‐2 microglia were transfected with the pcDNA3.1‐KLF3‐AS1, siRNA targeting ETV4 (si‐ETV4, RiboBio) and sh‐SIRT3 (RiboBio) using Lipofectamine 3000 reagent (Thermo Fisher Scientific). To model CIRI in vitro, BV‐2 microglia were exposed to oxygen‒glucose deprivation/reoxygenation (OGD/R). Briefly, cells were incubated in glucose‐free DMEM for 2 h under hypoxic conditions (94% N_2_, 1% O_2_, 5% CO_2_) at 37°C, followed by reoxygenation in complete DMEM under normoxia for 24 h. Control cells were maintained in standard culture conditions. BV‐2 cells were treated with BMSC‐derived exosomes at 10 µg/mL during the reoxygenation phase. BV‐2 cells were pre‐treated with vehicle or 3‐(1H‐1,2,3‐triazol‐4‐yl) pyridine (3‐TYP, Selleck) at 100 µM for 24 h and then treated with OGD/R and exosomes.

### 2.4 Clinical sample

CIRI patients and healthy controls were enrolled from Hunan Provincial People's Hospital (The First Affiliated Hospital of Hunan Normal University). Inclusion criteria: (1) age ≥ 18 years; (2) confirmed acute ischaemic stroke; (3) received reperfusion therapy within 24 h; and (4) evidence of reperfusion injury. Exclusion criteria: prior stroke, intracranial haemorrhage, severe organ diseases, malignancy, autoimmune diseases or pregnancy. Plasma was collected within 24 h post‐reperfusion and stored at −80°C. Participant characteristics are summarised in Table S1. All human plasma samples were obtained with written informed consent from all participants, and the study was approved by the Ethics Committee of Hunan Provincial People's Hospital (The First Affiliated Hospital of Hunan Normal University; approval number: 2024‐315).

### 2.5 Transmission electron microscopy

BV‐2 microglia were centrifugated at 10 000 *g* for 30 min to pellet cells. The supernatant was removed, and 2.5% glutaraldehyde solution was added into the cell pellets for 4 h of fixation at 4°C. Cell pellets were then washed and post‐fixed in 2% Osmium tetroxide for 1.5 h. After dehydration, cell pellets were consecutively incubated in acetone/embedding medium (1:2) for 1.5 h and embedding medium overnight. Ultrathin sections (70 nm) were prepared and stained with 2% uranyl acetate for 20 min and lead citrate for 10 min. Sections were washed and imaged under TEM. For quantitative analysis of mitochondrial morphology, at least 50 mitochondria per group were randomly selected from TEM micrographs obtained from three independent experiments. Mitochondrial swelling was defined by the presence of reduced matrix density, cristae separation, or matrix transparency. The percentage of swollen/damaged mitochondria was calculated as the ratio of swollen mitochondria to the total number of mitochondria counted per group. Autophagosome‐engulfed mitochondria were identified by the presence of double‐membrane structures enclosing mitochondria, and the number of such mitochondria was counted per group. All TEM images were analysed and scored independently by two investigators who were blinded to the group allocation. Quantification was performed using ImageJ software.

### 2.6 Mouse model of middle cerebral artery occlusion

C57BL/6 mice (male, 8‒10 weeks old, 25–30 g body weight) underwent middle cerebral artery occlusion (MCAO) modelling and were randomly divided into four groups (*n* = 8 per group): Sham, MCAO+PBS (phosphate buffered salin), MCAO+Exo^Mock^ and MCAO+Exo^KLF3‐AS1^. The MCAO procedure was performed as previously described.^21^ Briefly, mice were anaesthetised via intraperitoneal administration of 1% pentobarbital sodium, and a silicon‐coated 6‐0 nylon filament was introduced through the external carotid artery and advanced into the internal carotid artery to occlude the middle cerebral artery. After 1 h of ischaemia induction, the filament was retracted to permit cerebral reperfusion. Sham mice received same surgical procedure without occlusion. For mice in the MCAO/Exo^Mock^ and MCAO/Exo^KLF3‐AS1^, BMSC‐derived exosomes (10 µg) were intranasally injected into mice once daily for 3 consecutive days starting at 1 day after MCAO (day 1 to day 3 post surgery), and an equal volume of PBS was intranasally injected into mice in the MCAO+PBS group. Infract area was quantified at days 28 post‐MCAO procedure via 2,3,5‐triphenyltetrazolium chloride (TTC, Beyotime) staining. Animal procedures were conducted in accordance with the ARRIVE guidelines and approved by the Institutional Animal Care and Use Committee of Hunan Provincial People's Hospital (The First Affiliated Hospital of Hunan Normal University).

### 2.7 Neurobehavioural assessment

The modified neurological severity score (mNSS) was applied to evaluate neurological deficits at days 0, 1, 3, 7, 14 and 28. Severity was graded as follows: mild, 1–5; moderate, 6–10; and severe, 11–15. The grasping function was assessed through the foot fault test at days 0, 1, 3, 7, 14 and 28. Mice performed three consecutive traversals of a 1‐m horizontal ladder at controlled velocity, with digital video recording capturing locomotor performance. Footslip errors in the paretic limb were quantified as misplacement incidents relative to total steps. The coordination function was assessed by the rotarod test. Mice were trained for 3 days, and the time when mice fell off the rotarod before the MCAO procedure. After MCAO, the rotarod test was performed at postoperative days 3, 7, 14, and 28 using an accelerating rotarod (5‒40 rpm over 5 min).

### 2.8 Brain water content

Following euthanasia, cerebral tissues were immediately harvested for gravimetric analysis. Fresh mass determinations were performed prior to dehydration in a precision oven maintained at 80°C for 72 h, after which stable dry mass measurements were obtained. Brain water content was determined using the formula: [(fresh mass − desiccated mass)/fresh mass] × 100%.

### 2.9 Immunofluorescence and fluorescence in situ hybridisation staining

Mice were sacrificed, and the brains were harvested, dehydrated and embedded in Frozen Section Embedding Medium (Beyotime). Sections at 30 µm were coronally cut. Sections were fixed in acetone, rehydrated and blocked in 5% normal goat serum (Beyotime) for 1 h. Then, sections were stained with the anti‐CD16/32 (1:500, MA5‐47770, Thermo Fisher Scientific), anti‐CD206 (1:500, ab64693, Abcam), anti‐SIRT3 (1:100, PA5‐96406, Thermo Fisher Scientific) or anti‐Iba1 (1:1000, MA5‐50414, Thermo Fisher Scientific) antibody overnight. For cellular immunofluorescence (IF) staining, BV‐2 microglia were washed, fixed in 4% paraformaldehyde and permeabilised in 0.3% Triton X‐100. BV‐2 microglia were washed, blocked in 5% normal goat serum and incubated with the anti‐Inducible nitric oxide synthase (iNOS, 1:500, ab178945, Abcam), anti‐Arginase 1 (Arg‐1, 1:200, ab96183, Abcam), anti‐Iba1 (1:1000, MA5‐50414, Thermo Fisher Scientific) or anti‐ETV4 (1:500, 10684‐1‐AP, Proteictech) antibody overnight. Next day, sections and BV‐2 cells were washed and incubated with an Alexa Fluor 488 or 594‐conjugated secondary antibody (Jackson) for 1 h. The nuclei were stained with DAPI (Beyotime), and samples were mounted for imaging under Nikon Eclipse E600 Fluorescence Microscope (Nikon). For combined fluorescence in situ hybridisation (FISH) and IF staining, proteinase K (8 µg/mL, NEB) was added to treat cells and brain sections for 30 min, and samples were pre‐hybridised for 2 h. Cells and sections were hybridised with the Cy3‐conjugated KLF3‐AS1 probe mixture (3 oligonucleotides, 20 nt, 5′ Cy3; probe 1: Cy3‐AGCCGCCACACTTTCCCAAG, probe 2: Cy3‐GGGAGACGGGACTTACTTTG, probe 3: Cy3‐CGAGCGCAGCAATGTCAGGG; targeting the 5′ terminal, upstream middle, and middle regions of the KLF3‑AS1 transcript, respectively; RiboBio) at .2‒.5 µM at 58°C overnight. Cells and brain tissues were washed, blocked and incubated with the anti‐KAT6A (1:100, 15–827, ProSci), anti‐KDM4A (1:250, 702449, Thermo Fisher Scientific), anti‐NeuN (1:500, ab104225, Abcam), anti‐Iba1 (1:1000, MA5‐50414, Thermo Fisher Scientific) or anti‐GFAP (1:5000, ab7260, Abcam) for 1 h. Cells and sections were washed and incubated with Alexa Fluor 488‐conjugated secondary antibody (Jackson) for 1 h. The nuclei were stained with DAPI (Beyotime), and samples were mounted for imaging under Nikon Eclipse E600 Fluorescence Microscope.

### 2.10 JC‐1 and MitoTracker red staining

Mitochondrial membrane potential (MMP) was assessed by JC‐1 (AAT Bioquest) staining. JC‐1 exhibits MMP‐dependent accumulation in mitochondria, indicated by a fluorescence emission shift from green (JC‐1 monomers) to red (JC‐1 aggregates). Cells were stained with JC‐1 at 5 µM for 30 min and washed twice in pre‐warmed PBS. For MitoTracker Red staining, cells were stained with MitoTracker Red (M7512, Thermo Fisher Scientific) at 100 nM for 30 min washed twice in pre‐warmed PBS. Cells were imaged under Nikon Eclipse E600 Fluorescence Microscope. For mitochondrial length quantification, at least 50 individual mitochondria per group were randomly selected from three independent experiments.

### 2.11 Mitochondrial reactive oxygen species

Cells were stained with MitoSox Red (MCE) at 5 µM for 30 min and subsequently washed twice in PBS. Then, the nuclei were stained with DAPI (Beyotime). Cells were then imaged under Nikon Eclipse E600 Fluorescence Microscope.

### 2.12 Terminal deoxynucleotidyl transferase dUTP nick end labelling staining

Terminal deoxynucleotidyl transferase dUTP nick end labelling (TUNEL) staining was performed using Click‐iT Plus TUNEL Assay kit provided by Thermo Fisher Scientific. Briefly, brain frozen sections were fixed in 4% paraformaldehyde for 15 min and permeabilised in Proteinase K solution for 15 min. Then, samples were incubated in TdT reaction mixture buffer for 60 min and washed. The Click‐iT Plus reaction cocktail was prepared, and samples were incubated in this cocktail for 30 min. For neuronal co‐staining, sections were blocked and stained with rabbit anti‐NeuN antibody (1:500, ab104225, Abcam) for 1 h at room temperature. Sections were washed and stained with an Alexa Fluor 488‐conjugated goat anti‐rabbit immunoglobulin G (IgG) antibody (Jackson) for 1 h. After washing, the nuclei were stained with DAPI (Beyotime). Samples were mounted for imaging under Nikon Eclipse E600 Fluorescence Microscope. The fraction of TUNEL⁺NeuN⁺ apoptotic neurons was quantified as the percentage of total NeuN‐positive neurons in each field of view.

### 2.13 Cell Counting Kit‐8 and 5‐ethynyl‐2'‐deoxyuridine (EdU) incorporation assays

Cell proliferation was analysed by Cell Counting Kit‐8 (CCK‐8) and EdU incorporation assays. For CCK‐8 assay, cells (10^5^ cells/100 µL) were seeded in 96‐well plates, and 10 µL of CCK‐8 reagent (Beyotime) was added each well for 2 h of incubation at 37°C. The absorbance at 450 nm was recorded. For EdU incorporation, EdU was added into cells at 10 µM, and cells were incubated for 12 h. Click‐iT EdU Alexa Fluor 488 Imaging Kit (Thermo Fisher Scientific) was used to detect EdU‐positive cells. The nuclei were stained with DAPI, and cells were mounted for imaging under Nikon Eclipse E600 Fluorescence Microscope. The ratio of EdU‐positive cells to total DAPI‐positive cells was quantified.

### 2.14 Wound healing and Transwell assays

Cell migration was assessed by wound healing and Transwell assays. For wound healing assay, cells were seeded and allowed for a monolayer formation. The scratch was created on the monolayer using a 1 mL pipette, and cells were then incubated for additional 24 h. The scratch was imaged under Nikon Eclipse E600 Fluorescence Microscope to determine the wound healing. For Transwell assay, cells were seeded into the upper chamber of Transwell chamber (8‐µm pore, Corning), and complete medium was added into the lower chamber. Cells were then incubated for additional 24 h. Subsequently, cells that migrated into the lower chamber were fixed and stained with Crystal Violet Staining Solution (Beyotime) for 15 min. Cells were washed, air dried and imaged under Nikon Eclipse E600 Fluorescence Microscope.

### 2.15 Intracellular Ca^2+^ staining

To determine the levels of intracellular calcium, cells were stained with F03 calcium reagent (Bestbio) at 2 µM for 30 min at 37°C. After wash, cells were imaged under Nikon Eclipse E600 Fluorescence Microscope. The fluorescence intensity of calcium was quantified.

### 2.16 Thiazolyl Blue Tetrazolium Bromide (MTT) assay

Cells (10^5^ cells/100 µL) were seeded in 96‐well plates, and 10 µL of MTT reagent (Beyotime) was added each well for 4 h of incubation at 37°C. Insoluble formazan was redissolved in DMSO, and the absorbance at 540 nm was recorded.

### 2.17 Adenosine triphosphate measurement

Cells were lysed in Pierce Luciferase Lysis Buffer (Thermo Fisher Scientific) and centrifugated for collect the cell lysates. The levels of adenosine triphosphate (ATP) in the cell lysates were determined with ATP Determination Kit provided by Thermo Fisher Scientific according to the manufacturer's recommendations.

### 2.18 RNA extraction and quantitative real‐time PCR

Total RNA was extracted from BMSCs, BV‐2 microglia and brain tissues with RNeasy Mini Kit (QIAGEN) according the manufacturer's recommendations, and the quality of RNA was examined via gel electrophoresis. Nuclear and cytoplasmic RNAs were isolated from BMSCs using PARIS Kit (Thermo Fisher Scientific). For plasma samples, peripheral blood was collected into EDTA‐containing tubes and centrifuged at 3000 rpm for 10 min at 4°C to separate plasma, and total RNA was extracted directly from plasma using Quick‐cfDNA/RNA Serum & Plasma Kit (ZYMO RESEARCH). The expression of KLF3‐AS1, KAT6A, KDM4A, CD86, Tumour necrosis factor ‐ α (TNF‐α), Interleukin ‐ 1β (IL‐1β), Interleukin ‐ 6 (IL‐6), Arg‐1, Interleukin ‐ 10 (IL‐10), Transforming growth fcator ‐ β (TGF‐β), iNOS, SIRT3 and ETV4 were determined with real‐time quantitative PCR with SYBR Green (Bio‐Rad) and normalised to GAPDH. The 2^−∆∆Ct^ method was applied to calculate the fold change of gene expression. QuantiTect Reverse Transcription Kit (QIAGEN) was used to perform RNA reverse transcription to cDNA. The abundance of KLF3‐AS1, U6 and GAPDH was examined by quantitative real‐time PCR (qRT‐PCR). Information referring to the primers used in this study is shown in Table S2.

### 2.19 Western blotting

Protein was extracted from exosomes, BV‐2 microglia and brain tissues using ReadyPrep Protein Extraction Kit (Bio‐Rad) according to the manufacturer's recommendations. Nuclear and cytoplasmic protein fractions were extracted from BV‐2 microglia. Protein concentration was determined with BCA Protein Assay Kit (Abcam). Protein (20‒40 µg) was loaded into 10% sodium dodecyl sulfate polyacrylamide gel electrophoresis (SDS‒PAGE) for gel electrophoresis and transferred onto polyvinylidene fluoride (PVDF) membranes (Bio‐Rad). The membranes were blocked in 5% non‐fat milk solution and incubated with the rabbit anti‐mouse CD63 (1:1000, ab315108, Abcam), CD81 (1:2000, MA5‐32333, Thermo Fisher Scientific), TSG101 (1:1000, ab125011, Abcam), Calnexin (1:1000, ab22595, Abcam), iNOS (1:2000, ab283655, Abcam), Arg‐1 (1:1000, PA5‐85267, Thermo Fisher Scientific), SIRT3 (1:1000, PA5‐96406, Thermo Fisher Scientific), ETV4 (1:1000, PA5‐76825, Thermo Fisher Scientific), Lamin B1 (1:1000, ab16048, Abcam) or GAPDH (1:10 000, ab9485, Abcam) overnight. Membranes were washed and incubated with a goat anti‐rabbit IgG HRP secondary antibody (1:10 000, ab6721, Abcam) for 1 h. The signal was visualised with ECL substrate (Beyotime), and the band intensity was analysed with ImageJ software.

### 2.20 Chromatin immunoprecipitation

Cells were washed in PBS and crosslinked by adding 1% formaldehyde to a final concentration of 1% and incubated for 10 min at room temperaturesolution for 30 min. Crosslinking was quenched by adding glycine to a final concentration of 0.125 M for 5 min, and cells were washed and lysed in chromatin immunoprecipitation (ChIP) lysis buffer (Santa Cruz Biotechnology) on ice. Cells were centrifuged for collecting cell lysates, and the cell lysates were subjected to sonication to obtain DNA fragments (∼500 bp). Five micrograms of anti‐H3K27Ac (ab4729, Abcam), anti‐H3K27Me3 (9733, Cell Signaling Technology), anti‐KAT2B (3378, Cell Signaling Technology), anti‐KAT6A (CBMAB‐M1322‐FY, Creative Biolabs), anti‐GCN5 (MA5‐14884, Thermo Fisher Scientific), anti‐p300 (57625, Cell Signaling Technology), anti‐KDM1A (ab129195, Abcam), anti‐KDM4A (702449, Thermo Fisher Scientific), anti‐KDM5A (ab70892, Abcam), anti‐ETV4 (10684‐1‐AP, Proteictech) or normal IgG isotype was added into DNA fragments and incubated overnight at 4°C. Then, protein A/G magnetic beads (Thermo Fisher Scientific) were added, and the samples were incubated for additional 2 h. Samples were then decrosslinked, and DNA was recovered for analysing the enrichment at the promoter regions of KLF3‐AS1 or SIRT3 by qPCR.

### 2.21 RNA pull‐down assay

Biotin‐labelled sense and anti‐sense KLF3‐AS1 probes (RiboBio) were incubated with streptavidin magnetic beads (Thermo Fisher Scientific) for 2 h at 4°C. Cell lysates from BV‐2 microglia were prepared and incubated with probe‐coated beads overnight at 4°C. After washing, bound proteins were eluted and subjected to Western blotting with anti‐ETV4 antibody.

### 2.22 RNA immunoprecipitation assay

RNA immunoprecipitation (RIP) assay was performed using the Magna RIP RNA‐Binding Protein Immunoprecipitation Kit (Millipore). Briefly, BV‐2 microglia were lysed in RIP lysis buffer, and cell lysates were incubated with magnetic beads coated with anti‐ETV4 antibody (1:50, PA5‐76825, Thermo Fisher Scientific) or normal rabbit IgG as a negative control. After incubation, the beads were washed, and RNA was extracted from the immunoprecipitated complexes using TRIzol reagent. The enrichment of KLF3‐AS1 was analysed by qRT‐PCR and normalised to IgG control.

### 2.23 Dual luciferase reporter assay

Wildtype or mutant SIRT3 promoter (SIRT3‐WT or SIRT3‐MUT) was cloned into the pGL3 vector (Promega). BV‐2 cells were co‐transfected with pcDNA3.1‐ETV4 (oe‐ETV4) and SIRT3‐WT or SIRT3‐MUT. The empty pcDNA3.1 vector (oe‐NC) was used as the negative control. After 48 h, the luciferase activity was measured with Dual‐Glo Luciferase System (Promega).

### 2.24 Statistical analysis

Experimental data were generated from triplicate biological replicates and statistically represented as mean ± SD through GraphPad Prism 9.0. Bivariate comparisons employed Student's *t*‐test, whereas multigroup analyses utilised one‐way ANOVA with Tukey post hoc test. *p* < .05 was statistically significant.

## 3 RESULTS

### 3.1 KLF3‐AS1 promoted BMSC viability, proliferation and migration

To investigate the role of KLF3‐AS1 in BMSCs, we examined the subcellular localisation of KLF3‐AS1 within BMSCs. Bioinformatic analysis utilising the LncATLAS database^22^ showed that KLF3‐AS1 exhibited predominant nuclear localisation (Figure 1A). Moreover, KLF3‐AS1 showed a bimodal subcellular distribution, localised in both cytoplasmic and nuclear fractions, with predominant nuclear enrichment, which was confirmed by RNA‐FISH staining (Figure 1B,C). Then, we overexpressed KLF3‐AS1 in BMSCs (Figure 1D). We found that overexpression of KLF3‐AS1 increased BMSC viability (Figure 1E). EdU incorporation assays showed that overexpression of KLF3‐AS1 enhanced EdU incorporation, suggesting that BMSC proliferation was promoted by KLF3‐AS1 overexpression (Figure 1F). Furthermore, through TUNEL, wound healing and Transwell assays, we found that overexpression of KLF3‐AS1 inhibited BMSC apoptosis but promoted its migratory capacity (Figure 1G–I). Collectively, KLF3‐AS1 enhanced BMSC viability, proliferation and migration but inhibited its apoptosis.

**FIGURE 1:**
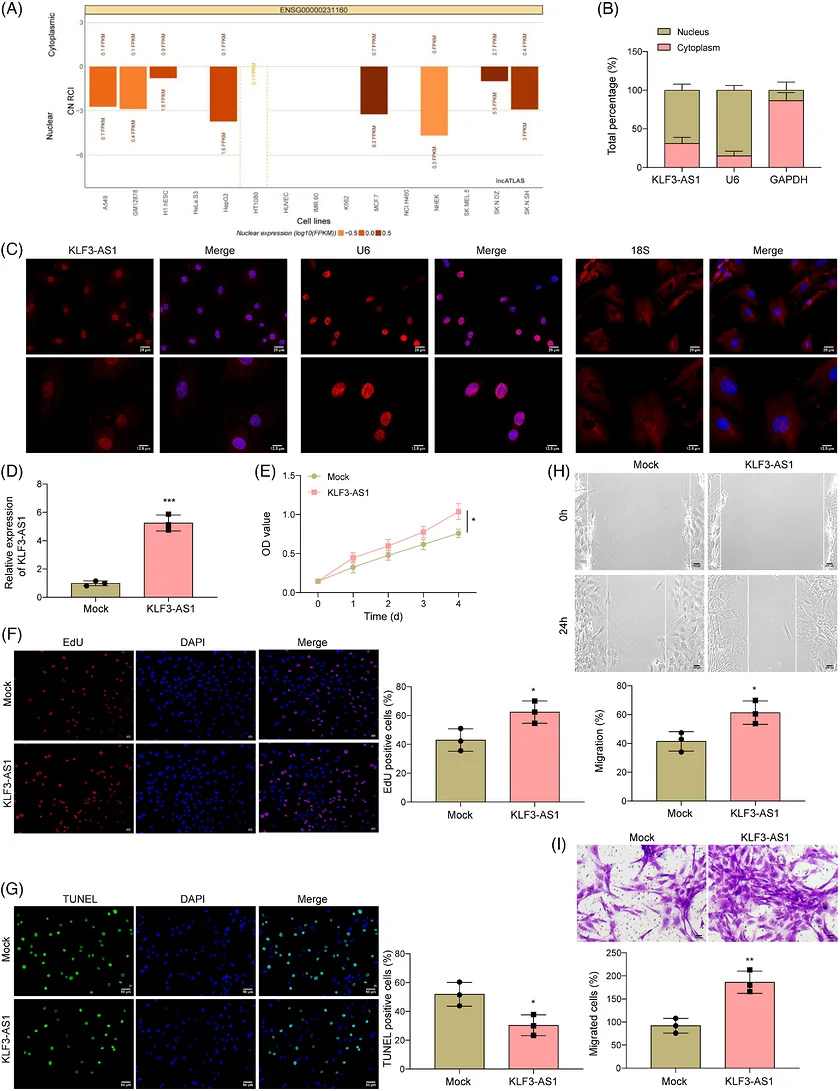
KLF3‐AS1 promoted bone marrow mesenchymal stem cell (BMSC) viability, proliferation and migration. KLF3‐AS1 was overexpressed in BMSCs through KLF3‐AS1 transfection. BMSCs were divided into Mock and KLF3‐AS1 groups. (A) The LncATLAS database predicted that KLF3‐AS1 was primarily localised to the nucleus. (B) The cytoplasmic and nuclear fractions were separated, and the abundance of KLF3‐AS1, U6 and GAPDH was examined by quantitative real‐time PCR (qRT‐PCR) (*n* = 3). (C) RNA‐fluorescence in situ hybridisation (FISH) staining for KLF3‐AS1 (red). The nuclei (blue) were stained with DAPI. Scale bar: 25 µm. (D) qRT‐PCR analysis of KLF3‐AS1 (*n* = 3). (E) Cell viability was examined with MTT on days 1, 2, 3, and 4 (*n* = 3). (F) EdU (red) incorporation assays for analysing BMSC proliferation. The nuclei (blue) were stained with DAPI. Scale bar: 50 µm. (G) Cell apoptosis was determined with TUNEL (green) staining. The nuclei (blue) were stained with DAPI. Scale bar: 50 µm. (H and I) Wound healing and Transwell assays were performed to evaluate BMSC migration. Scale bar: 100 µm. ^*^ *p *< .05, ^**^ *p *< .01 and ^***^ *p *< .001.

### 3.2 Increased H3K27Ac and decreased H3K27Me3 at the KLF3‐AS1 promoter were associated with KAT6A and KDM4A in BMSCs

Bioinformatic analysis^23^ annotated the full range of the KLF3‐AS1 promoter region, defined as the 2000 bp genomic fragment spanning chr4: 38 664 000–38 666 000 (GRCh38/hg38) surrounding the transcription start site (TSS), which covers the core epigenetic regulatory region with abundant H3K27ac and H3K27me3 modifications, and supplemented detailed H3K27me3 modification distribution information, together with H3K27ac data, to comprehensively exhibit the epigenetic modification landscape of the KLF3‐AS1 promoter (Figure 2A). ChIP‐qPCR analysis revealed significant enrichment of H3K27ac at the KLF3‐AS1 promoter region. In contrast, H3K27me3 exhibited modest but reproducible enrichment at the same locus, with signal intensity substantially lower than that of H3K27ac (Figure 2B,C). These complementary histone modification profiles suggest that the KLF3‐AS1 promoter may be under dual, functionally opposing epigenetic regulation‐activation‐associated H3K27ac and repression‐associated H3K27me3‐potentially enabling context‐dependent bidirectional control of transcription.Treatment with C646, a selective inhibitor of p300/CBP histone acetyltransferases, reduced KLF3‐AS1 expression (Figure 2D), indicating that global histone acetylation is required for KLF3‐AS1 transcription. Then, we screened histone lysine acetyltransferases and demethylases that bound to KLF3‐AS1 promoter via ChIP assays, and found that KAT6A and KDM4A were the primary histone lysine acetyltransferase and demethylase binding to KLF3‐AS1 promoter (Figure 2E,F). IF combined with RNA‐FISH staining exhibited that both KLF3‐AS1 and KAT6A or KDM4A predominantly localised in the nuclei of BMSCs (Figure 2G). KAT6A was knocked down in BMSCs, and KLF3‐AS1 was downregulated by KAT6A knockdown (Figure 2H). The H3K27Ac antibody‐mediated enrichment of KLF3‐AS1 promoter was impaired by KAT6A knockdown (Figure 2I). Also, overexpression of KDM4A reduced KLF3‐AS1 expression and decreased H3K27Me3 enrichment at the KLF3‐AS1 promoter as detected by ChIP with an H3K27Me3 antibody (Figure 2J,K). These data suggested that KAT6A and KDM4A were associated with the increased H3K27Ac and decreased H3K27Me3 at the KLF3‐AS1 promoter in BMSCs.

**FIGURE 2:**
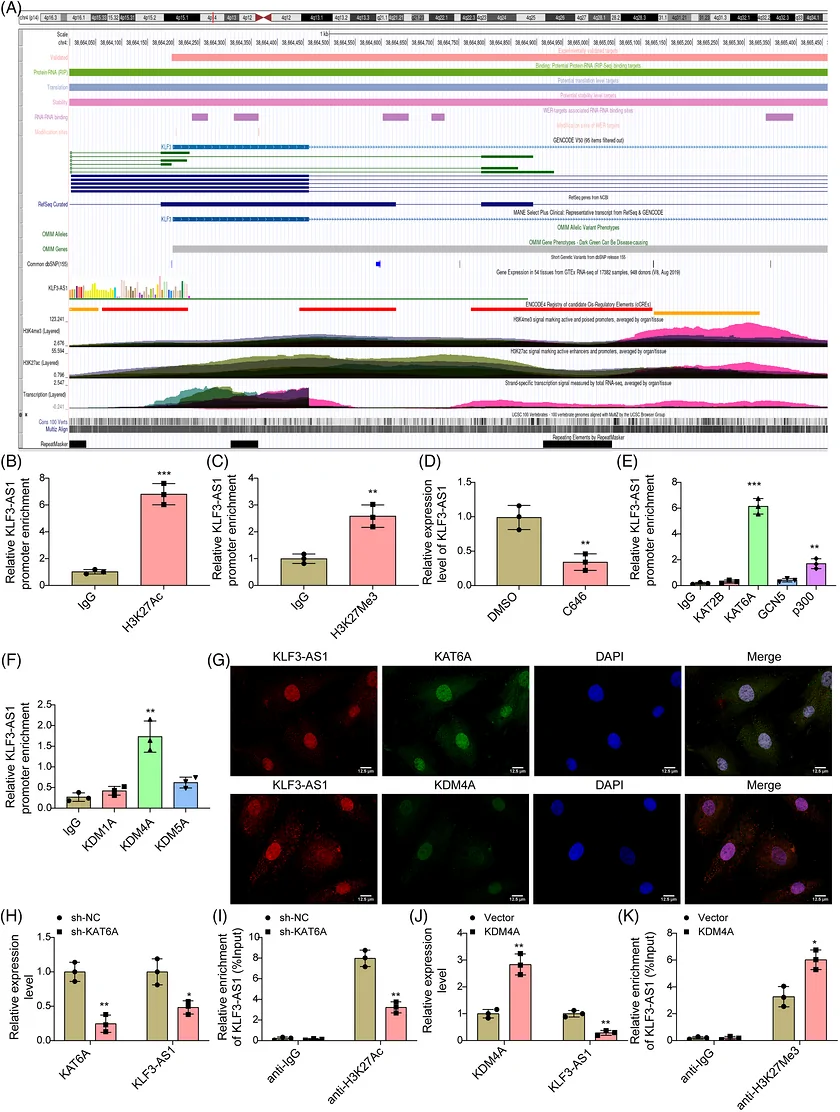
Increased H3K27Ac but decreased H3K27Me3 at the KLF3‐AS1 promoter in bone marrow mesenchymal stem cells (BMSCs). (A) Predictive analysis by the UCSC Genome Browser (https://genome.ucsc.edu/) of KLF3‐AS1 and the enrichment of histone acetylation and methylation in its promoter region. (B and C) Chromatin immunoprecipitation (ChIP)‐qPCR assays were performed to determine the enrichment of KLF3‐AS1 promoter by the antibody against H3K27Ac or H3K27Me3 in BMSCs (*n* = 3). (D) Quantitative real‐time PCR (qRT‐PCR) analysis of KLF3‐AS1 expression in BMSCs treated with DMSO or C646 (*n* = 3). (E and F) ChIP‐qPCR assays were performed to determine the enrichment of KLF3‐AS1 promoter by the antibody against KAT2B, KAT6A, GCN5, p300, KDM1A, KDM4A or KDM5A (*n* = 3). (G) The localisation of KLF3‐AS1 (red), KAT6A (green) or KDM4A (green) was examined by combined immunofluorescence (IF) and fluorescence in situ hybridisation (FISH) staining. The nuclei (blue) were stained with DAPI. Scale bar: 12.5 µm. (H) qRT‐PCR analysis of KAT6A and KLF3‐AS1 in BMSCs transfected with sh‐NC or sh‐KAT6A (*n* = 3). (I) ChIP‐qPCR analysis of the level of H3K27Ac of the KLF3‐AS1 promoter in BMSCs transfected with sh‐NC or sh‐KAT6A (*n* = 3). (J) qRT‐PCR analysis of KDM4A and KLF3‐AS1 in BMSCs transfected with vector of KDM4A (*n* = 3). (K) ChIP‐qPCR analysis of the level of H3K27Me3 of the KLF3‐AS1 promoter in BMSCs transfected with vector of KDM4A (*n* = 3). ^*^ *p *< .05, ^**^ *p *< .01 and ^***^ *p *< .001.

### 3.3 Exosomal KLF3‐AS1 derived from BMSCs ameliorated MCAO‐induced neurological injury in mice

As KLF3‐AS1 can be packaged in exosomes to improve myocardial ischaemia‒reperfusion injury,^24^ we determined to analyse the effects of exosomal KLF3‐AS1 derived from BMSCs on CIRI in a mouse model of MCAO. BMSC‐derived exosomes (Exo^Mock^ and Exo^KLF3‐AS1^) exhibited typical cup‐shaped morphology under TEM (Figure S1A), expressed exosomal markers CD63, CD81 and TSG101 but not Calnexin (Figure S1B), and showed a mean particle size of approximately 120 nm by NTA (Figure S1C). KLF3‐AS1 abundance was increased in Exo^Mock^ and further increased in Exo^KLF3‐AS1^ (Figure S1D). The mouse model of MCAO was established, and the experimental procedure was designed as shown in Figure 3A. Mice were divided into Sham, MCAO + PBS, MCAO + Exo^Mock^ and MCAO + Exo^KLF3‐AS1^ groups. The mNSS score and partial placement ratio were low in the Exo^Mock^ group compared to that in the PBS group, and they were further reduced by Exo^KLF3‐AS1^ (Figure 3B,C), implying that Exo^Mock^ and Exo^KLF3‐AS1^ improved MCAO‐induced neurological deficit and grasping function. The rotarod test exhibited that, compared to mice in the PBS group, mice in the Exo^Mock^ group could stay longer on the rotarod, and the time was further prolonged in mice from the Exo^KLF3‐AS1^ group (Figure 3D). The increased infarction area and water content caused by MCAO in the brain were reduced by Exo^Mock^ and further reduced by Exo^KLF3‐AS1^ (Figure 3E,F). In addition, MCAO‐induced apoptosis of NeuN‐positive neurons was mitigated by Exo^Mock^, and this protective effect was further enhanced by Exo^KLF3‐AS1^ (Figure 3G). Thus, exosomal KLF3‐AS1 derived from BMSCs contributed to the amelioration of mice from MCAO‐induced neurological damage.

**FIGURE 3:**
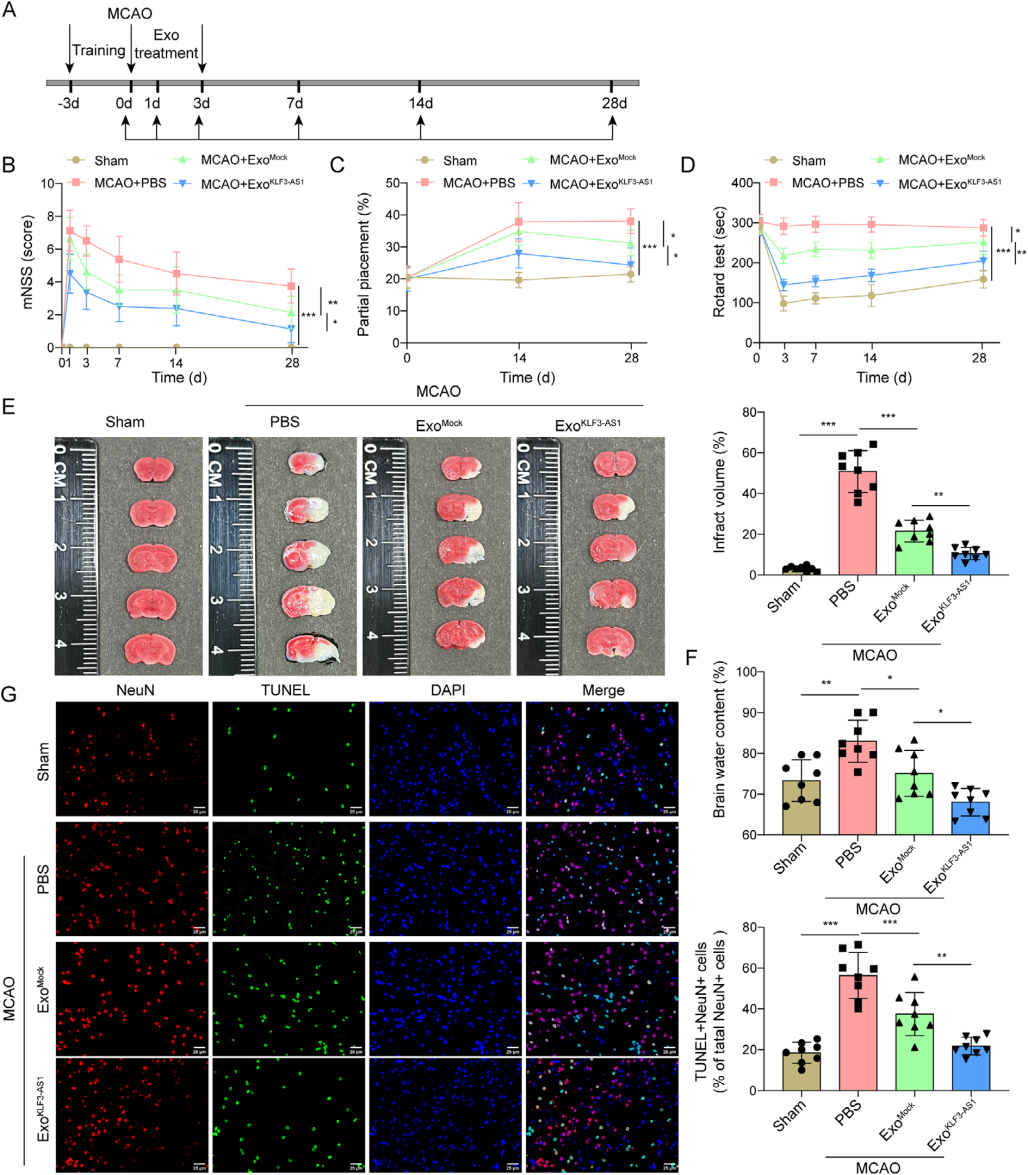
Exosomal KLF3‐AS1 derived from bone marrow mesenchymal stem cells (BMSCs) ameliorated middle cerebral artery occlusion (MCAO)‐induced neurological injury in mice. (A) Flow chart of animal experiment design. BMSC‐derived exosomes were intranasally administrated on days 0, 1, 2 and 3 after MCAO. (B) The neurological function was evaluated by modified neurological severity score (mNSS) scoring (*n* = 8). (C) The grasping function was assessed by foot fault test (*n* = 8). (D) The rotarod test was performed to assess the coordination function of mice (*n* = 8). (E) Representative 2,3,5‐triphenyltetrazolium chloride (TTC) staining images of coronal brain sections from mice. Infract volume was quantified (*n* = 8). (F) Brain water content was examined (*n* = 8). (G) Representative images of double immunostaining of NeuN (red) and TUNEL (green). The nuclei (blue) were stained with DAPI. Scale bar: 25 µm. NeuN and TUNEL‐double positive cells were quantified. ^*^ *p *< .05, ^**^ *p *< .01 and ^***^ *p *< .001.

### 3.4 KLF3‐AS1‐enriched exosomes derived from BMSCs shifted microglia towards an M2‑like phenotype in MCAO mice

Given that previous studies have demonstrated that intranasal administration of exosomes can effectively deliver exosomes to neurons and microglia in the brain,^21^, ^25^ we hypothesise that exosomal KLF3‐AS1 may also be internalised by neurons and microglia. Double staining of KLF3‐AS1 and the neuron marker NeuN, the microglial marker Iba1 or the astrocyte marker GFAP was performed. We observed a co‐localisation of KLF3‐AS1 and Iba1, while no co‐localisation of KLF3‐AS1 with NeuN or GFAP was observed (Figure 4A). PKH26‐labelled BMSC‐Exos were intranasally administered at 24 h after MCAO, and the internalisation of BMSC‐Exos by Iba1+ microglial cells was detected at 6 h after BMSC‐Exo administration (Figure 4B). Moreover, DiI‐labelled BMSC‐Exos were added into BV‐2 cells, and we observed the internalisation of BMSC‐Exos in BV‐2 cells (Figure 4C). These observations suggested that exosomes derived from BMSCs predominantly entered microglia in mice. We further analysed whether the internalisation of BMSC‐Exos regulated microglia polarisation. IF staining showed that Iba1‐postive cells were significantly increased by MCAO (Figure 4D–G). MCAO significantly elevated CD16/32 and Iba1‐double positive M1‐polarised microglia but moderately increased CD206 and Iba1‐double positive M2‐like microglia (Figure 4D–G). However, Exo^Mock^ administration reduced CD16/32 and Iba1‐double positive M1‐like microglia but increased CD206 and Iba1‐double positive M2‐like microglia, and these effects on microglial polarisation were further enhanced by Exo^KLF3‐AS1^ (Figure 4D–G). Furthermore, MCAO‐induced expression of M1 markers CD86, TNF‐α, IL‐1β and IL‐6 was reduced by Exo^Mock^ and further reduced by Exo^KLF3‐AS1^ (Figure 4H). No significant change in the expression of M2 markers Arg‐1 and IL‐10 was observed, but TGF‐β was downregulated after MCAO procedure (Figure 4I). The expression of these M2‐associated markers was promoted by Exo^Mock^ and further enhanced by Exo^KLF3‐AS1^ (Figure 4I). Moreover, iNOS was significantly upregulated, while Arg‐1 expression was slightly increased after MCAO procedure (Figure 4J). Exo^Mock^ reduced iNOS expression but promoted Arg‐1 expression, and these regulatory effects on the expression of iNOS and Arg‐1 were further enhanced by KLF3‐AS1‐enriched exosomes (Figure 4J). To conclude, BMSC‐derived exosomes entered microglia and switched microglia to the M2‐like phenotype in the brain of MCAO mice, and KLF3‐AS1‐enriched exosomes further enhanced the effect on microglial polarisation.

**FIGURE 4:**
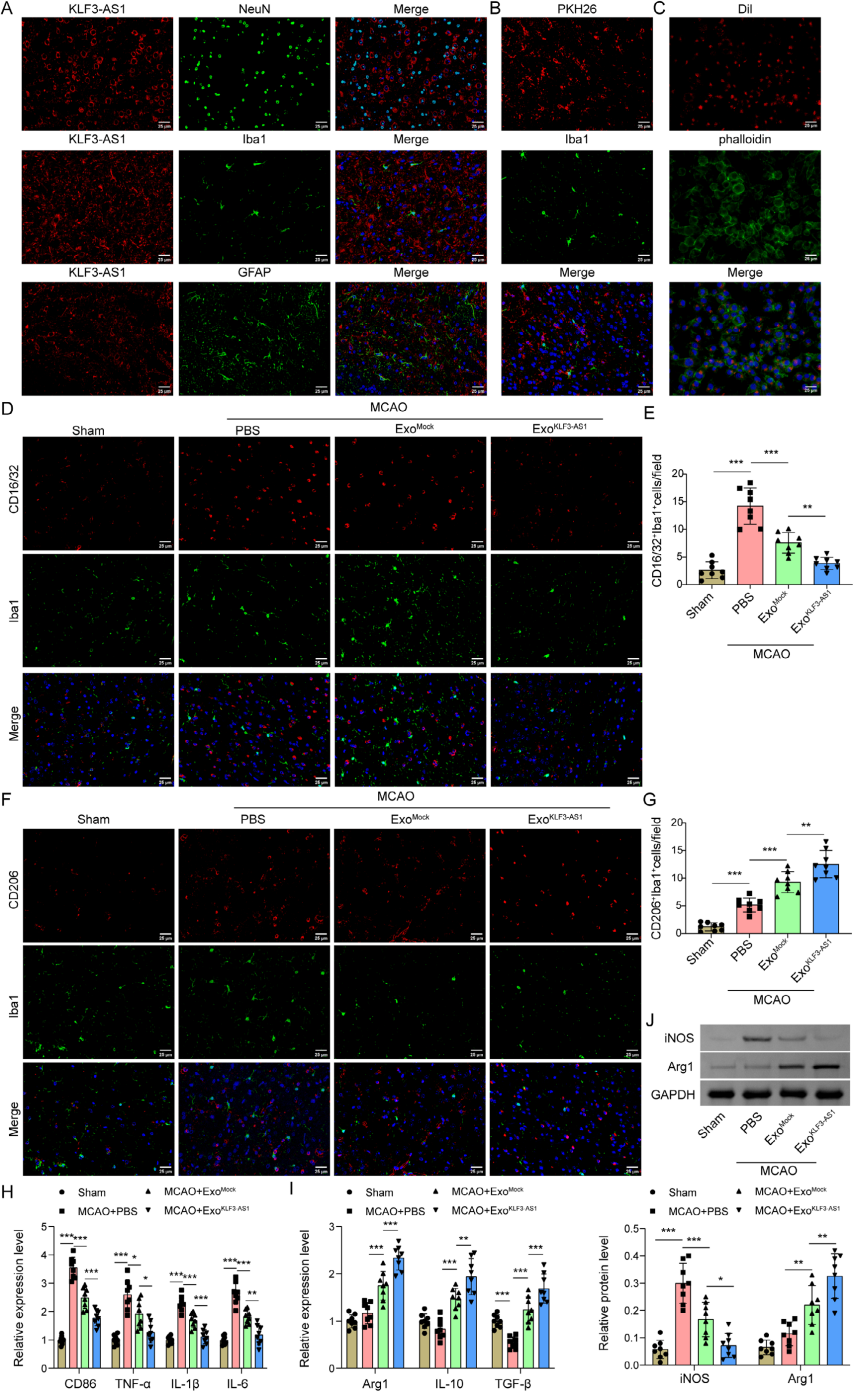
KLF3‐AS1 enriched exosomes derived from bone marrow mesenchymal stem cells (BMSCs) shifted microglia towards an M2‑like phenotype in middle cerebral artery occlusion (MCAO) mice. (A) Co‐localisation of KLF3‐AS1 (red) and NeuN (green), Iba1 (green) or GFAP (green) was examined by combined immunofluorescence (IF) and fluorescence in situ hybridisation (FISH) staining. The nuclei (blue) were stained with DAPI. Scale bar: 25 µm. (B) PKH26‐labelled BMSC‐derived exosomes (red) were intranasally injected at 24 h after MCAO. IF staining of Iba1 (green) was performed to assess the internalisation of BMSC‐derived exosomes by microglial cells. Scale bar: 25 µm. (C) DiI‐labelled BMSC‐derived exosomes (red) were added into BV‐2 cells, and fluorescent imaging showed the internalisation of exosomes within BV‐2 cells. Actin (green) was stained with phalloidin. Scale bar: 25 µm. (D‒G) IF staining of Iba1 (green) and CD16/32 (red) or CD206 (red). Iba1+ CD16/32+ and Iba1+ CD206+ double positive cells were quantified (*n* = 8 per group). The nuclei (blue) were stained with DAPI. Scale bar: 25 µm. (H and I) Quantitative real‐time PCR (qRT‐PCR) analysis of CD86, TNF‐α, IL‐1β, IL‐6, Arg‐1, IL‐10 and TGF‐β (*n* = 8 per group). (J) iNOS and Arg‐1 were detected by Western blotting. ^*^ *p *< .05, ^**^ *p *< .01 and ^***^ *p *< .001.

### 3.5 KLF3‐AS1‐enriched exosomes derived from BMSCs shifted microglia towards an M2‑like phenotype in OGD/R‐treated BV‐2 cells

To further investigate whether KLF3‐AS1‐enriched exosomes regulated microglia polarisation, we treated BV‐2 cells with OGD/R in the presence or absence of exosome treatment and assessed the change of microglia polarisation phenotypes. The viability of BV‐2 cells was impaired by OGD/R treatment, and Exo^Mock^ improved BV‐2 cell viability (Figure 5A). BV‐2 cells in the OGD/R + Exo^KLF3‐AS1^ group showed highest viability (Figure 5A). OGD/R‐induced expression of CD86, iNOS, TNF‐α, IL‐1β and IL‐6 was suppressed by Exo^Mock^ and further inhibited by Exo^KLF3‐AS1^ (Figure 5B). In contrast, the expression of Arg‐1 was elevated by OGD/R, while OGD/R reduced IL‐10 and TGF‐β expression in BV‐2 cells (Figure 5C). The expression of Arg‐1, IL‐10 and TGF‐β was enhanced by Exo^Mock^ and further enhanced by Exo^KLF3‐AS1^ (Figure 5C). Co‐staining of Iba1 and iNOS or Arg‐1 showed that significantly increased iNOS and Iba1‐double positive cells were reduced by Exo^Mock^ and further reduced by Exo^KLF3‐AS1^, while the number of Arg‐1 and Iba1‐double positive cells increased slightly following OGD/R injury. Treatment with either Exo^Mock^ or Exo^KLF3‐AS1^ treatment significantly increased the abundance of these double‑positive cells, and this effect was particularly pronounced in the reinforced KLF3‑AS1 group. (Figure 5D). These results implied that BMSC‐derived KLF3‐AS1‐enriched exosomes shifted microglia towards an M2‑like phenotype in BV‐2 cells.

**FIGURE 5:**
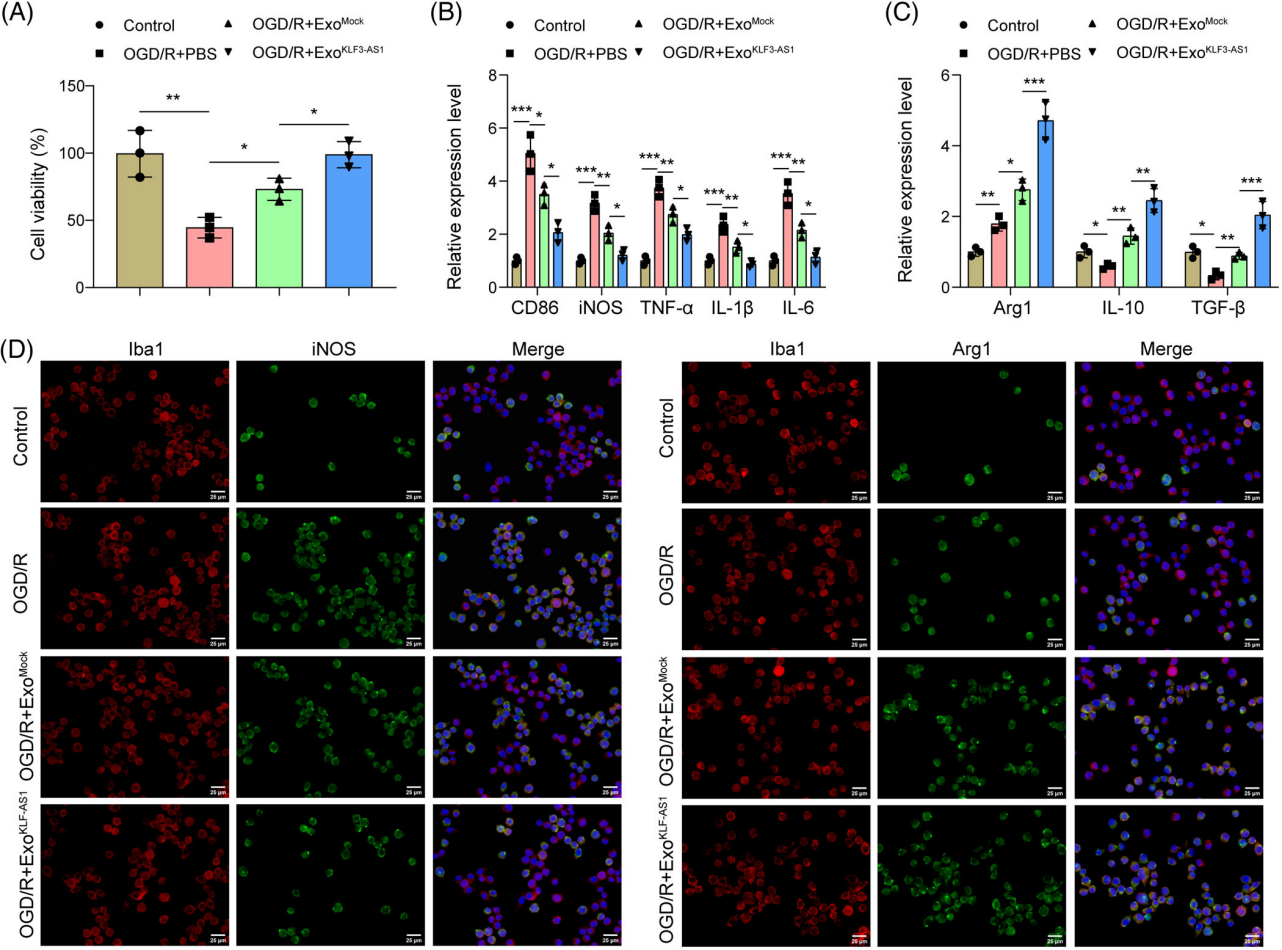
KLF3‐AS1‐enriched exosomes derived from bone marrow mesenchymal stem cells (BMSCs) shifted microglia towards an M2‑like phenotype in oxygen‒glucose deprivation/reoxygenation (OGD/R)‐treated BV‐2 cells. BV‐2 cells were treated with OGD/R in the presence or absence of exosomes and divided into Control, OGD/R, OGD/R + Exo^Mock^ and OGD/R + Exo^KLF3‐AS1^ groups. (A) Cell viability was examined with Cell Counting Kit‐8 (CCK‐8) (*n* = 3). (B and C) Quantitative real‐time PCR (qRT‐PCR) analysis of CD86, iNOS, TNF‐α, IL‐1β, IL‐6, Arg‐1, IL‐10 and TGF‐β (*n* = 3). (D) Immunofluorescence (IF) staining of Iba1 (red) and iNOS (green) or Arg‐1 (green). The nuclei (blue) were stained with DAPI. Scale bar: 25 µm. ^*^ *p *< .05, ^**^ *p *< .01 and ^***^ *p *< .001.

### 3.6 BMSC‐derived KLF3‐AS1‐enriched exosomes ameliorated OGD/R‐induced oxidative stress and mitochondrial dysfunction and upregulated SIRT3 in microglia

Given that mitochondrial dysfunction, encompassing mitochondrial DNA damage, metabolic deficits and quality control disturbances, precedes microglial activation and subsequent inflammatory responses,^26^ we then investigated the mitochondrial structure, oxidative stress and functional alterations in BV‐2 cells after OGD/R and exosome treatment. BV‐2 cells were divided into Control, OGD/R, OGD/R + Exo^Mock^ and OGD/R + Exo^KLF3‐AS1^ groups. TEM revealed severe mitochondrial swelling, cristae disruption and shortening in OGD/R‐challenged BV2 cells, which was partially reversed by Exo^Mock^ and further attenuated by Exo^KLF3‐AS1^, and quantitative analysis confirmed that the increased mitochondrial length and restored number of mitochondrial cristae per mitochondrion were displayed upon the treatment of Exo^KLF3‐AS1^, accompanied by an increased fraction of mitophagosome‐encapsulated mitochondria (Figure 6A). Moreover, MitoTracker staining showed that the mitochondrial length was reduced in the OGD/R group, which was partially revered by Exo^Mock^ and further reversed by Exo^KLF3‐AS1^ (Figure 6A). Increased intracellular Ca^2+^ and mitochondrial reactive oxygen species (ROS) in OGD/R‐treated BV‐2 cells were reduced by Exo^Mock^ and further reduced by Exo^KLF3‐AS1^ (Figure 6B,C). Given that increased Ca^2+^ and mitochondrial ROS are two important factors that exacerbate mitochondrial dysfunction, we analysed MMP to further evaluate mitochondrial function. We found that the ratio of JC‐1 monomers and aggregates was dramatically increased by OGD/R treatment, which was partially reversed by Exo^Mock^ and further reduced by Exo^KLF3‐AS1^ (Figure 6D), suggesting that BMSC‐derived KLF3‐AS1‐enriched exosomes improved MMP in OGD/R‐treated BV‐2 cells. Furthermore, the decreased level of ATP in OGD/R‐treated BV‐2 cells was partially reversed in the OGD/R + Exo^Mock^ group, and Exo^KLF3‐AS1^ further recovered the level of ATP (Figure 6E). As Sirtuin 3 (SIRT3) has been reported to alleviate neuroinflammation by improving mitochondrial function in BV‐2 cells,^27^ we determined to examine the expression of SIRT3. SIRT3 was downregulated in OGD/R‐treated BV‐2 cells, but its expression was increased by Exo^Mock^ and further increased by Exo^KLF3‐AS1^ (Figure 6F,G). Moreover, Iba1 and SIRT3‐double positive cells in the brain from MCAO mice were decreased, which was increased by Exo^Mock^ and further increased by Exo^KLF3‐AS1^ (Figure S2). To conclude, BMSC‐derived KLF3‐AS1‐enriched exosomes ameliorated oxidative stress and mitochondrial dysfunction and upregulated SIRT3 in microglia.

**FIGURE 6:**
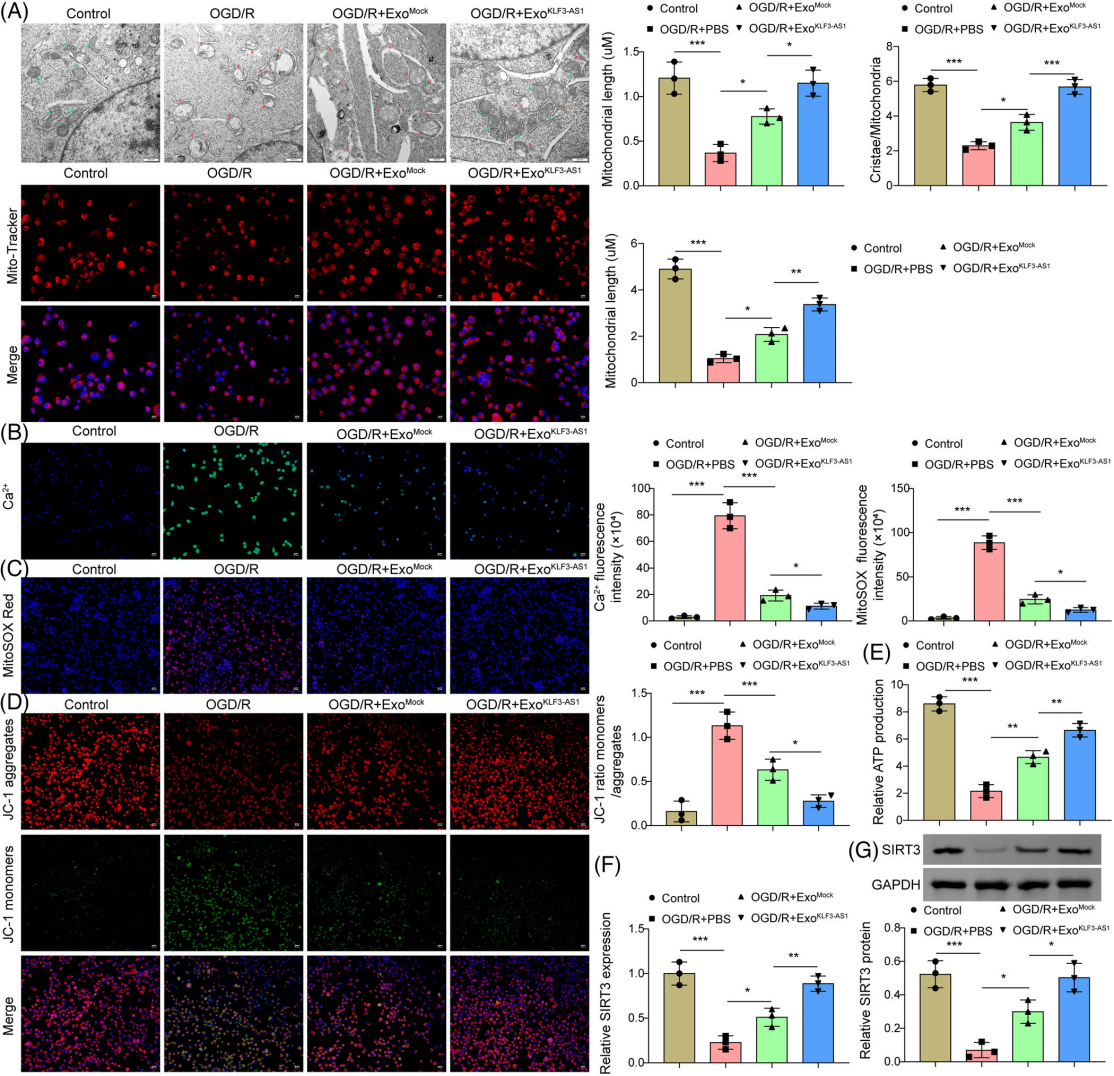
Bone marrow mesenchymal stem cell (BMSC)‐derived KLF3‐AS1‐enriched exosomes ameliorated oxygen‒glucose deprivation/reoxygenation (OGD/R)‐induced oxidative stress and mitochondrial dysfunction and upregulated Silent Information Regulator 3 (SIRT3) in microglia. BV‐2 cells were divided into Control, OGD/R, OGD/R + Exo^Mock^ and OGD/R + Exo^KLF3‐AS1^ groups. (A) Mitochondria was examined by transmission electron microscopy (TEM) and MitoTracker Red staining. In TEM images, black arrow indicated mitochondrial swelling, red arrow indicated normal mitochondria and yellow arrow indicated autophagosomal engulfment of mitochondria. Mitochondrial length and cristae number were quantified. (B) F03 staining was performed to analyse intracellular Ca^2+^ (green) levels (*n* = 3). Scale bar: 50 µm. (C) Mitochondrial reactive oxygen species (ROS) (red) was examined by MitoSox Red staining (*n* = 3). Scale bar: 50 µm. (D) Measurement of mitochondrial membrane potential by JC‐1 staining (*n* = 3). JC‐1 aggregates, red; JC‐1 monomers, green. Scale bar: 50 µm. (E) Adenosine triphosphate (ATP) levels were quantified in BV‐2 cells (*n* = 3). (F and G) Quantitative real‐time PCR (qRT‐PCR) and Western blotting analysis of SIRT3 (*n* = 3). ^*^ *p *< .05, ^**^ *p *< .01 and ^***^ *p *< .001.

### 3.7 KLF3‐AS1 activated SIRT3 expression by facilitating ETV4 nuclear translocation in microglia

KLF3‐AS1 recruits ETV4 to upregulate SIRT1 expression, thus alleviating cerebral ischaemia/reperfusion injury.^14^ Thus, we overexpressed KLF3‐AS1 in BV‐2 cells and examined the expression of ETV4. Overexpression of KLF3‐AS1 did not affect the expression of ETV4 in BV‐2 cells (Figure 7A). IF staining revealed that ETV4 was distributed in both the nucleus and cytoplasm, with lower abundance in the nucleus in the Mock group (Figure 7B). However, overexpression of KLF3‐AS1 led to a predominant nuclear localisation of ETV4 (Figure 7B). Consistently, western blotting analysis of nuclear and cytoplasmic fractions showed that overexpression of KLF3‐AS1 significantly increased the nuclear accumulation of ETV4, as evidenced by a marked elevation in the nuclear/cytoplasmic ratio of ETV4 (Figure 7A). To explore whether KLF3‐AS1 directly interacts with ETV4, we performed RNA pull‐down assay using biotin‐labelled KLF3‐AS1 sense and anti‐sense probes. ETV4 was pulled down by the KLF3‐AS1 sense probe, whereas no ETV4 signal was detected with the anti‐sense probe (Figure S3A). To further validate this interaction, we performed RIP assay using an anti‐ETV4 antibody. KLF3‐AS1 was significantly enriched in the ETV4‐immunoprecipitated fraction compared to the IgG control (Figure S3B). These data confirmed a direct physical interaction between KLF3‐AS1 and ETV4. A potential binding site for ETV4 in the promoter of SIRT3 was predicted via JASPAR (Figure 7C). We constructed wildtype and mutant SIRT3 luciferase reporters (Figure 7D). Overexpression of ETV4 significantly enhanced the luciferase activity of the wildtype SIRT3 reporter, but the luciferase activity of the mutant SIRT3 reporter was not affected (Figure 7E). ChIP assays confirmed that the SIRT3 promoter could be enriched by a ETV4 antibody (Figure 7F), and overexpression of KLF3‐AS1 promoted the enrichment of ETV4 in the promoter of SIRT3 (Figure S4). Additionally, overexpression of ETV4 significantly upregulated SIRT3 in BV‐2 cells (Figure 7G,H). These findings suggested that KLF3‐AS1 promoted the nuclear translocation of ETV4 to enhance SIRT3 expression in microglia. To establish the causal role of ETV4 in KLF3‑AS1‑mediated SIRT3 upregulation and neuroprotection, we performed rescue experiments in BV‑2 microglia. Cells were divided into Control, OGD/R + Mock + si‑NC, OGD/R + KLF3‑AS1 + si‑NC, and OGD/R + KLF3‑AS1 + si‑ETV4 groups. KLF3‑AS1 overexpression improved OGD/R‑impaired cell viability, which was reversed by ETV4 knockdown (Figure 7I). Moreover, KLF3‑AS1 overexpression suppressed M1 markers (CD86, iNOS, TNF‑α, IL‑1β, IL‑6) and promoted M2 markers (Arg‑1, IL‑10, TGF‑β), while ETV4 knockdown abolished these regulatory effects (Figure 7J). KLF3‑AS1 promoted microglial M1‑to‑M2 polarisation, which was largely reversed by ETV4 knockdown (Figure 7K). JC‑1 staining revealed that KLF3‑AS1 overexpression restored OGD/R‑induced MMP loss, and this effect was abrogated by ETV4 knockdown (Figure 7L). Furthermore, both qRT‑PCR and Western blotting confirmed that KLF3‑AS1‑induced SIRT3 upregulation was ETV4‑dependent (Figure 7M,N). Collectively, these results implied that ETV4 was required for KLF3‑AS1‑mediated SIRT3 upregulation, mitochondrial protection and anti‑inflammatory microglial polarisation.

**FIGURE 7:**
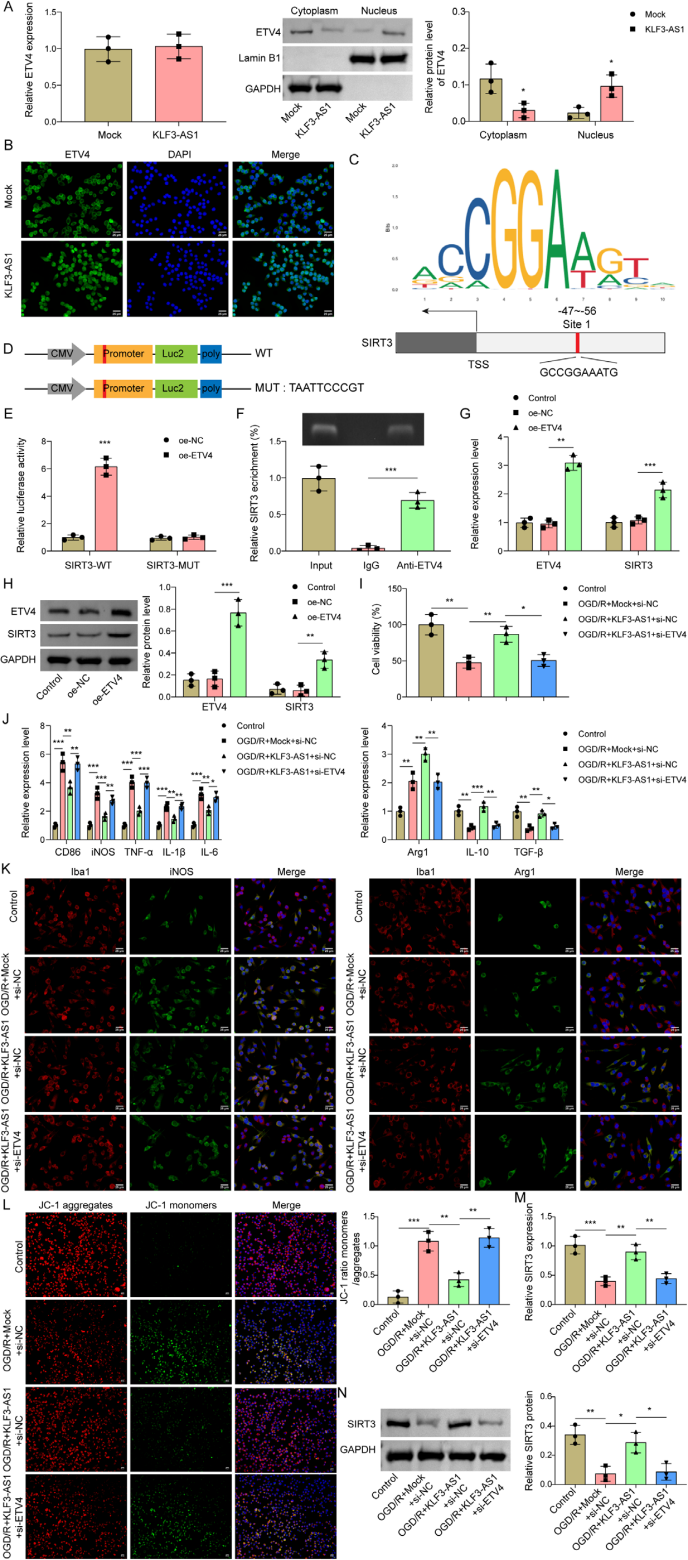
KLF3‐AS1 activated Silent Information Regulator 3 (SIRT3) expression by facilitating ETS Variant Transcription Factor 4 (ETV4) nuclear translocation in microglia. (A) Quantitative real‐time PCR (qRT‐PCR) analysis of ETV4 in BV‐2 cells transfected with Mock or KLF3‐AS1 (*n* = 3). (B) Immunofluorescence (IF) staining of ETV4 (green) in BV‐2 cells transfected with Mock or KLF3‐AS1. The nuclei (blue) were stained with DAPI. Scale bar: 25 µm. Nuclear and cytoplasmic fractionation followed by Western blotting analysis of ETV4 in BV‐2 cells transfected with Mock or KLF3‐AS1. Lamin B1 and GAPDH were used as nuclear and cytoplasmic fractionation controls, respectively. (C) The binding site for ETV4 in SIRT3 promoter was predicted via JASPAR. (D) Wildtype and mutant SIRT3 luciferase reporters were constructed. (E) BV‐2 cells were transfected with oe‐ETV4 and SIRT3‐WT or SIRT3‐Mut luciferase reporter, and the luciferase activity was determined (*n* = 3). (F) Chromatin immunoprecipitation (ChIP)‐qPCR analysis of the binding of ETV4 to SIRT3 promoter (*n* = 3). (G and H) qRT‐PCR and Western blotting analysis of ETV4 and SIRT3 (*n* = 3). Cells were divided into Control, oxygen‒glucose deprivation/reoxygenation (OGD/R) + Mock + si‑NC, OGD/R + KLF3‑AS1 + si‑NC and OGD/R + KLF3‑AS1 + si‑ETV4 groups. (I) Cell viability was examined with Cell Counting Kit‐8 (CCK‐8) (*n* = 3). (J) qRT‐PCR analysis of CD86, iNOS, TNF‐α, IL‐1β, IL‐6, Arg‐1, IL‐10 and TGF‐β (*n* = 3). (K) IF staining of Iba1 (red) and iNOS (green) or Arg‐1 (green). The nuclei (blue) were stained with DAPI. Scale bar: 25 µm. (L). Measurement of mitochondrial membrane potential by JC‐1 staining (*n* = 3). JC‐1 aggregates, red; JC‐1 monomers, green. Scale bar: 50 µm. (M and N) qRT‐PCR and Western blotting analysis of SIRT3 (*n* = 3). ^*^ *p *< .05, ^**^ *p *< .01 and ^***^ *p *< .001.

### 3.8 Inhibition of SIRT3 reversed exosomal KLF3‐AS1‐mediated protective effects on OGD/R‐treated microglia

To determine whether KLF3‐AS1‐mediated protective effects on OGD/R‐treated microglia were dependent on SIRT3, we overexpressed KLF3‐AS1 in BV‐2 cells under OGD/R and simultaneously knocked down SIRT3 using sh‐SIRT3 or inhibited it with 3‐TYP, a SIRT3 inhibitor. KLF3‐AS1 improved cell viability, which was reversed by SIRT3 depletion or inhibition (Figure S5A). KLF3‐AS1‐mediated suppression of M1 markers (CD86, iNOS, TNF‐α, IL‐1β, IL‐6) and elevation of M2 markers (Arg‐1, IL‐10, TGF‐β) were abolished by SIRT3 knockdown or inhibition (Figure S5B). IF confirmed that KLF3‐AS1‐induced M1‐to‐M2 polarisation was SIRT3‐dependent (Figure S5C). Additionally, SIRT3 loss eliminated KLF3‐AS1‐mediated reductions in mitochondrial ROS (Figure S5D), restoration of MMP (Figure S5E) and recovery of ATP levels (Figure S5F). qRT‐PCR and Western blotting further confirmed that SIRT3 upregulation by KLF3‐AS1 was effectively blocked by sh‐SIRT3 or 3‐TYP (Figure S5G,H). Collectively, these results suggested that SIRT3 was the essential downstream effector of KLF3‐AS1 in protecting against OGD/R‐induced mitochondrial dysfunction and inflammatory polarisation. We also found that CIRI patients showed decreased expression of KLF3‐AS1 and SIRT3, and the expression of KLF3‐AS1 and SIRT3 were positively correlated in patients through Spearman correlation coefficient analysis (Figure S6A‒C), suggesting the implication of KLF3‐AS1 and SIRT3 in cerebral ischaemia reperfusion injury of patients.

## 4 DISCUSSION

CIRI represents a critical pathological phenomenon exacerbating neuronal damage rather than restoring normal function, contributing significantly to morbidity and mortality in ischaemic stroke.^28^ The dual‐phase pathogenesis involves initial ischaemic hypoxia such as ATP depletion, ionic imbalance and excitotoxicity followed by reperfusion‐triggered oxidative stress, inflammatory cascades, and mitochondrial dysfunction, collectively activating apoptotic and necroptotic pathways.^29^, ^30^ Current therapeutic strategies focus on ROS scavengers, anti‐inflammatory agents and blood‒brain barrier stabilisers. Emerging approaches include hypothermia therapy, stem cell‐derived exosome‐mediated repair and mitochondrial‐targeted antioxidants.^31^, ^32^ Despite advances, clinical translation remains challenged by the complexity of CIRI pathogenesis, underscoring the need for seeking novel therapeutic targets and strategies. This study demonstrates that KAT6A and KDM4A differentially regulate KLF3‐AS1 transcription through opposing epigenetic modifications at its promoter: KAT6A promotes histone acetylation, whereas KDM4A mediates histone demethylation. Consequently, KLF3‐AS1 expression is bidirectionally controlled. Notably, elevated KLF3‐AS1 carried by BMSC‐derived exosomes facilitated nuclear translocation of ETV4, thereby enhancing SIRT3 transcription (Figure 8). This KLF3‐AS1/ETV4/SIRT3 axis improved mitochondrial function, promoted microglial polarization toward the anti‐inflammatory M2 phenotype, and ultimately attenuated CIRI.

**FIGURE 8:**
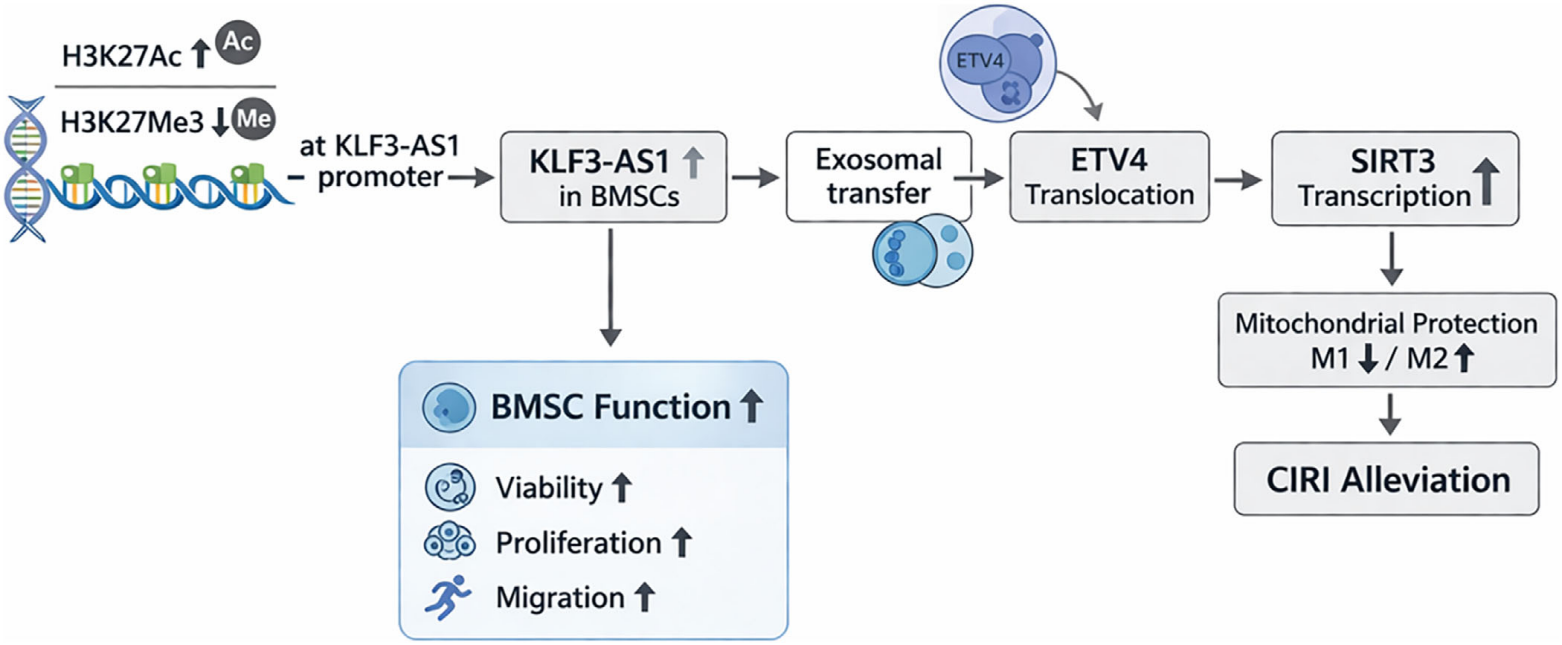
Schematic illustration of this study. Bone marrow mesenchymal stem cell (BMSC)‐derived exosomes deliver KLF3‐AS1 into microglia, where KLF3‐AS1 upregulates Silent Information Regulator 3 (SIRT3) expression. Elevated SIRT3 subsequently promotes microglial polarisation from the pro‐inflammatory M1 phenotype towards the anti‐inflammatory M2 phenotype, while simultaneously attenuating oxidative stress and inflammatory responses.

BMSC transplantation facilitates neuronal and glial differentiation, promoting cerebral tissue repair and functional recovery in CIRI.^33^ KLF3‐AS1 encapsulated in BMSC‐derived exosomes ameliorate neurological deficits and endothelial dysfunction following subarachnoid haemorrhage.^13^ Notably, BMSC‐derived exosomes exert therapeutic effects against CIRI via delivering KLF3‐AS1.^14^, ^15^ Enhancing BMSC viability and inhibiting apoptosis during transplantation significantly reduces infarct volume, mitigates cellular apoptosis, delays vascular atrophy and ultimately improves CIRI.^34^ However, whether KLF3‐AS1 improves CIRI by mediating BMSC functionality remains unclear. Here, we found that overexpression of KLF3‐AS1 enhanced BMSC viability, proliferation and migration and inhibited BMSC apoptosis. Thus, upregulating KLF3‐AS1 expression in BMSCs to modulate BMSC functionality represents a viable therapeutic strategy for CIRI. Aberrant expression of lncRNAs arises from transcriptional activation, a process modulated by histone acetylation and methylation. The histone acetyltransferase KAT6A shows reduced expression in senescent BMSCs, and overexpression of KAT6A restores stemness properties and enhances osteogenic differentiation capacity in aged BMSCs.^35^ Overexpression of the demethylase KDM4A potentiates BMSC osteogenic differentiation.^36^ The histone acetylation and methylation at the KLF3‐AS1 promoter remain largely unknown. Intriguingly, KAT6A and KDM4A are associated with histone acetylation and demethylation at the KLF3‐AS1 promoter, synergistically upregulating KLF3‐AS1 expression in BMSCs. However, the underlying mechanisms remain to be fully elucidated.

BMSC‐derived exosomes circumvent risks associated with whole‐BMSC transplantation, such as tumourigenicity and immune rejection.^37^, ^38^ Growing evidence has revealed that exosomes improve CIRI via various mechanisms such as cell apoptosis inhibition and M1/M2 polarisation regulation.^39^ Our findings indicated that BMSC‐derived exosomes engineered to carry KLF3‐AS1 significantly ameliorated neurological deficits in MCAO‐induced CIRI. Building on prior evidence that exosomal KLF3‐AS1 mitigates ischaemia‒reperfusion injury,^14^, ^15^ this study further supports its therapeutic potential to CIRI. The protective effects observed in the KLF3‐AS1‐enriched exosome group on improving mNSS scores, rotarod endurance, infarct volume reduction, cerebral oedema attenuation and neuronal apoptosis suppression suggests a positive correlation between KLF3‐AS1 cargo levels and functional recovery, aligning with emerging recognition of exosomal lncRNAs as important regulators of CIRI.^40^, ^41^ While the enhanced protection against NeuN‐positive neuronal apoptosis in the KLF3‐AS1‐enriched exosome group points to specific anti‐apoptotic programming mediated by KLF3‐AS1, the precise mechanisms remain to be fully mapped. Our study underscores the importance of KLF3‐AS1 cargo engineering in developing exosome‐based therapeutic approaches.

While our data confirm the therapeutic potential of KLF3‐AS1‐loaded exosomes over naïve BMSC‐derived exosomes, the mechanisms remain to be fully mapped. Microglia are the primary resident immune cells in the brain. Studies have shown that environmental changes during CIRI can drive microglial polarisation, particularly metabolic alterations that induce the excessive activation of pro‐inflammatory microglia, leading to secondary injury.^42^  Numerous studies demonstrate that mediating the shift of microglia from the pro‐inflammatory M1 phenotype to the anti‐inflammatory M2 phenotype ameliorates CIRI,^43^, ^44^ making this polarisation process one of promising therapeutic targets for CIRI. The disruption of metabolic pathways during CIRI primarily occurs in mitochondria, and mitochondrial dysfunction is recognised as a hallmark of CIRI.^45^ Importantly, mitochondrial dysfunction alters the immune phenotype of microglia, thereby hindering the activation of M2 microglia while favouring the M1 pro‐inflammatory phenotype.^46^ Our study elucidates a novel neuroprotective mechanism through which KLF3‐AS1‐enriched exosomes derived from BMSCs restore mitochondrial homeostasis and reprogram microglial phenotype in CIRI. Both in vivo and in vitro approaches indicate that exosomal KLF3‐AS1 preferentially targets microglia rather than neurons or astrocytes and mitigates mitochondrial swelling, Ca^2+^ overload, ROS accumulation and MMP collapse while restoring ATP production, effectively shifting microglia phenotypes from M1‐like to M2‐like. The targeting of mitochondrial homeostasis and microglial polarisation positions KLF3‐AS1‐enriched exosomes as a therapeutic strategy for CIRI and other neuroinflammatory disorders. Notably, the neuroprotection observed in this study may involve both direct and indirect mechanisms. Previous studies have demonstrated that BMSC‐derived exosomal KLF3‐AS1 directly promotes neuronal viability and suppresses apoptosis,^14^ and ETV4 directly inhibits neuronal ferroptosis via the YBX1/GPX4 axis.^19^ Meanwhile, our finding that exosomal KLF3‐AS1 shifts microglia towards the M2 phenotype would indirectly reduce neuroinflammation and oxidative stress, creating a protective microenvironment. Thus, we propose a dual mechanism: direct neuronal protection via KLF3‐AS1/ETV4 pathways and indirect protection via microglial M2 polarisation. Distinguishing these contributions warrants future investigation. However, the binary M1/M2 classification of microglial polarisation is an oversimplification of the complex and dynamic activation states that microglia exhibit in CIRI. Our analysis relied on classical surface markers and did not capture the full heterogeneity of microglial responses. Advanced approaches such as flow cytometry, bulk RNA sequencing or single‐cell sequencing would provide a more comprehensive characterisation of microglial phenotypic changes and functional reprogramming, which we plan to pursue in future studies.

Our previous studies have suggested that SIRT3 alleviates neuroinflammation by ameliorating mitochondrial dysfunction in BV‐2 microglia.^27^ Additionally, SIRT3 mitigates CIRI by improving mitochondrial dysfunction in neurons.^47^ Thus, we hypothesised that exosomal KLF3‐AS1 might be involved in mitochondrial homeostasis and microglial polarisation by targeting SIRT3. Indeed, we found downregulation of SIRT3 in OGD/R‐treated microglia and MCAO mice, and KLF3‐AS1‐enriched exosomes restored its expression. The functional necessity of SIRT3 modulation is underscored by 3‐TYP‐mediated SIRT3 inhibition experiments, which abolished exosomal KLF3‐AS1‐mediated protective effects on mitochondrial dysregulation and microglial polarisation, positioning SIRT3 as the linkage connecting exosomal KLF3‐AS1 to neuroprotective effects in CIRI. Previous studies have reported that ETV4 is required for gene expression,^48^, ^49^, ^50^ and our previous work indicated that KLF3‐AS1 recruits ETV4 to transcriptionally activate Sirt1, thereby enhancing autophagy to alleviate cerebral ischaemia/reperfusion injury.^14^ In this study, we found that KLF3‐AS1 enhanced ETV4 nuclear translocation. Mechanistically, RNA pull‐down and RIP assays confirmed a direct physical interaction between KLF3‐AS1 and ETV4, consistent with our prior observations. By this interaction, ETV4 directly bound to the SIRT3 promoter to activate SIRT3 transcription in microglia. Importantly, ETV4 knockdown effectively abrogated the protective effects of KLF3‐AS1 overexpression on mitochondrial function and microglial polarisation, confirming that ETV4 is required for KLF3‐AS1‐mediated SIRT3 upregulation and neuroprotection. These findings delineate a novel KLF3‐AS1/ETV4/SIRT3 regulatory axis that orchestrates mitochondrial homeostasis and microglial polarisation under ischaemia‒reperfusion stress, extending our previous understanding of the KLF3‐AS1/ETV4 axis beyond SIRT1‐mediated autophagy to include SIRT3‐mediated mitochondrial regulation.

In conclusion, KLF3‐AS1 significantly enhances BMSC viability, proliferation and migration while suppressing apoptosis, and KLF3‐AS1‐enriched exosomes derived from BMSCs ameliorate CIRI by maintaining mitochondrial function and inhibiting inflammatory microglial polarisation through the ETV4/SIRT3 axis. Our study elucidates the mechanisms by which exosomal KLF3‐AS1 derived from BMSCs ameliorates CIRI and identify novel therapeutic targets and cell‐free strategies for CIRI management. Clinically, the positive correlation between KLF3‐AS1 and SIRT3 in CIRI patients validates the translational relevance of this axis, suggesting its dysregulation contributes to human ischaemia‒reperfusion pathophysiology. Importantly, our findings establish a conceptually novel mechanism that extends beyond previous reports on KLF3‐AS1 in CIRI. Earlier studies demonstrated that BMSC‐derived exosomal KLF3‐AS1 protects against CIRI through ETV4/SIRT1‐mediated autophagy or miR‐206/USP22/SIRT1‐mediated protein stabilisation.^14^, ^15^ In contrast, our study reveals a distinct pathway involving SIRT3, a mitochondrial‐localised sirtuin governing oxidative stress and energy homeostasis, rather than SIRT1. Furthermore, while ETV4 has been recently reported to suppress neuronal ferroptosis via the YBX1/GPX4 axis in CIRI, we uncover a novel role of ETV4 in regulating microglial mitochondrial function and inflammatory polarisation through SIRT3 transcriptional activation. Additionally, we provide the first evidence that KAT6A‐mediated histone acetylation and KDM4A‐mediated histone demethylation at the KLF3‐AS1 promoter epigenetically regulate its expression in BMSCs. Although we identified KAT6A and KDM4A as key enzymes associated with the histone modification changes at the KLF3‐AS1 promoter, rescue experiments restoring KLF3‐AS1 expression after KAT6A knockdown or KDM4A manipulation would further strengthen the causal link between these epigenetic modifications and KLF3‐AS1‐mediated biological effects. While KDM4A is classically a H3K9me3/H3K36me3 demethylase, evidence shows it also possesses H3K27me3 activity.^51^ We acknowledge that KDM6A/B are the canonical H3K27me3 demethylases and may also participate in this regulation.

However, several limitations of this study should be acknowledged. First, the precise molecular mechanism by which KLF3‐AS1 promotes ETV4 nuclear translocation, whether it acts as a scaffold, induces conformational changes, or recruits specific transport machinery, remains to be fully elucidated and warrants further investigation. Second, our clinical validation is limited by the small sample size. Larger, multicentre cohorts are required to confirm the diagnostic and prognostic value of circulating KLF3‐AS1 and SIRT3. Third, we have not systematically quantified exosome biodistribution, delivery efficiency, or targeting specificity in vivo, which will be critical for optimising therapeutic regimens. Fourth, the M1/M2 microglial polarisation framework, while useful, is an oversimplification of the complex and dynamic microglial activation states that occur in CIRI, and future studies using single‐cell approaches are needed to capture this heterogeneity. Fifth, the translational applicability of exosomal KLF3‐AS1 therapy requires further evaluation regarding long‐term safety, immunogenicity, storage stability and GMP‐compatible production. Our future studies will focus on addressing these gaps to advance towards clinical translation.

Several translational considerations warrant discussion for clinical application of BMSC‑derived exosomal KLF3‑AS1 in ischaemic stroke. First, exosome engineering, including surface modification with brain‑targeting ligands and cargo loading optimisation, can enhance delivery specificity and therapeutic efficacy. Second, dosing and delivery route optimisation are critical; intranasal administration offers a non‑invasive route that bypasses the blood‒brain barrier, although systematic dose‒response studies are needed to establish the optimal window. Third, safety and stability profiles of MSC‑derived exosomes appear favourable, yet long‑term safety data, off‑target effects and storage stability require further characterisation. Finally, clinical application faces challenges in standardisation, scalable GMP‑compatible production and rigorous validation. Despite these challenges, BMSC‑derived exosomal KLF3‑AS1 represents a promising cell‑free therapeutic strategy for CIRI.

## AUTHOR CONTRIBUTIONS

Qian Li conceived and designed the experiments. Qian Li and Xiaowei Xie wrote the manuscript. Qian Li, Jie Chen and Yu Cao performed all experiments. Xiaowei Xie and Dingzhou Zhou collected data and performed the statistical analysis. All the authors read and approved the final version of the manuscript and agree to take responsibility for the published article.

## CONFLICT OF INTEREST STATEMENT

The authors declare they have no conflicts of interest.

## ETHICS STATEMENT

Animal procedures were conducted in accordance with the ARRIVE guidelines and approved by the Institutional Animal Care and Use Committee of Hunan Provincial People's Hospital (The First Affiliated Hospital of Hunan Normal University).

## Supporting information



Supporting information

## Data Availability

The data that support the findings of this study are available from the corresponding author upon reasonable request.
