# Supplementary material for: Intranuclear localisation of KLF3‐AS1 under the balance of histone acetylation and methylation in BMSCs ameliorates cerebral ischaemia‒reperfusion injury by preserving microglial mitochondrial function and inhibiting inflammatory polarisation
Source: Clin Transl Med. 2026 Oct 31;16(11):e70870. doi: 10.1002/ctm2.70870 (PMC13723223; doi:10.1002/ctm2.70870)
Supplement: Supplementary file 1 [file CTM2-16-e70870-s001.docx]

**Supplementary materials**

**Supplementary figure legends**

**Supplementary Figure 1. Characterization of BMSC‑derived exosomes.**  Exosomes were isolated from vector or KLF3-AS1-transfected BMSCs (Exo^Mock^ and Exo^KLF3-AS1^). (A) Representative TEM image of exosomes. Scale bar, 100 nm. (B) Western blotting analysis of exosomal markers (CD63, CD81, TSG101) and the endoplasmic reticulum marker Calnexin. (C) Particle size distribution determined by NTA. (D) qRT‑PCR analysis of KLF3‑AS1 in BMSCs, Exo^Mock^, and Exo^KLF3‑AS1^. ***P* < 0.01.

**Supplementary Figure 2. KLF3-AS1-enriched exosomes upregulated SIRT3 in microglia.** Representative images of IF staining of Iba1 (Green) and SIRT3 (Red) in the brain tissues from mice in the Sham, MCAO/PBS, MCAO/Exo^Mock^ and MCAO/Exo^KLF3-AS1^ group. The nuclei (Blue) were stained with DAPI. Scale bar, 25 μm. ***P* < 0.01 and ****P* < 0.001.

**Supplementary Figure 3. KLF3‑AS1 directly interacted with ETV4.**  (A) RNA pull‑down assay using biotin‑labeled KLF3‑AS1 sense or anti‑sense probes, followed by Western blotting with anti‑ETV4 antibody. Input (10% of cell lysate) served as positive control. (B) RIP assay using anti‑ETV4 antibody or IgG control, followed by qRT‑PCR analysis of KLF3‑AS1 enrichment. **P* < 0.05.

**Supplementary Figure 4. Overexpression of KLF3-AS1 facilitated the binding of ETV4 to the SIRT3 promoter.** ChIP-qPCR analysis of the binding of ETV4 to the SIRT3 promoter (n = 3). **P* < 0.05, ***P* < 0.01 and ****P* < 0.001.

**Supplementary Figure 5. SIRT3 was required for KLF3‑AS1‑mediated neuroprotection in OGD/R‑treated microglia.** BV-2 cells were divided into Control, OGD/R + Mock + sh-NC, OGD/R + KLF3-AS1 + sh-NC, OGD/R + KLF3-AS1 + sh-SIRT3, and OGD/R + KLF3-AS1 + 3-TYP groups. (A) Cell viability (n = 3). (B) qRT‑PCR analysis of M1 markers (CD86, iNOS, TNF‑α, IL‑1β, IL‑6) and M2 markers (Arg‑1, IL‑10, TGF‑β) (n = 3). (C) IF staining of Iba1 (Red) with iNOS (Green) or Agr-1 (Green). Nuclei were counterstained with DAPI (Blue). Scale bar, 25 μm. (D) Mitochondrial ROS was detected by MitoSOX Red staining (Red). Scale bar, 50 μm. (E) Mitochondrial membrane potential was measured by JC‑1 staining. Scale bar, 50 μm. (F) ATP levels (n = 3). (G and H) qRT‑PCR and Western blotting analysis of SIRT3. **P* < 0.05, ***P* < 0.01 and ****P* < 0.001.

**Supplementary Figure 6. The expression of KLF3-AS1 and SIRT3 were positively correlated in patients with CIRI.** The peripheral blood was collected from healthy volunteers and patients with CIRI, which was prepared for plasma separation. (A and B) qRT-PCR analysis of KLF3-AS1 and SIRT3 in the plasma samples (healthy volunteers, n = 20; patients, n = 15). (C) Spearman correlation analysis of the expression of KLF3-AS1 and SIRT3 in patients with CIRI. ****P* < 0.001.

**Supplementary Table 1. Participant characteristics.**

| **Characteristic** | **CIRI (n = 15)** | **Control (n = 20)** |
| --- | --- | --- |
| Age (years), | 75.4±12.8 (45–96) | 64.6±9.7 (35–76) |
| Sex, male/female | 7/8 | 11/9 |
| **Previous medical history** |  |  |
| Atrial fibrillation, n (%) | 8 (53.3%) | – |
| Hypertension, n (%) | 12(80%) | – |
| Hyperlipidemia, n (%) | 9 (60%) | – |
| Diabetes, n (%) | 0 (0%) | – |
| Smoking, n (%) | 6 (40%) | – |
| Blood sampling time for onset (h), Mean ± SD | 12.5±7.15 | – |
| NIHSS, median (min, max) | 4 (0–22) | – |

**Supplementary Table 2. qRT-PCR primers.**

| Mouse KLF3-AS1 | Forward: 5′-AATGAGTGCGTGGAGGAAAT-3′ |
| --- | --- |
|  | Reverse: 5′-CCTGGGCAACAGAGTGAGAC-3′ |
| Human KLF3-AS1 | Forward: 5′-CTGTAGGCGCGCTCTTTCTTT-3′ |
|  | Reverse: 5′-TCCGACCAAAGTTTGCCAAG-3′ |
| Human KAT6A | Forward: 5′-TGAGTGGATTTTGGAGGCCAT-3′ |
|  | Reverse: 5′-GACCAAATCATCAATGTCATCATAGG-3′ |
| Human KDM4A | Forward: 5′-GCTTCTGAGTCTGAAACTCTGAATCC-3′ |
|  | Reverse: 5′-GACCAAATCATCAATGTCATCATAGG-3′ |
| Mouse CD86 | Forward: 5′-CATGGGCTTGGCAATCCTTA-3′ |
|  | Reverse: 5′-AAATGGGCACGGCAGATATG-3′ |
| Mouse TNF-α | Forward: 5′-GACGTGGAACTGGCAGAAGAG-3′ |
|  | Reverse: 5′-TTGGTGGTTTGTGAGTGTGAG-3′ |
| Mouse IL-1β | Forward: 5′-AAGGAGAACCAAGCAACGACAAAA-3′ |
|  | Reverse: 5′-TGGGGAACTCTGCAGACTCAAACT-3′ |
| Mouse IL-6 | Forward: 5′-GGTCCAGTTGCCTTCTCCC-3′ |
|  | Reverse: 5′-GCAACAAGGAACACCACGG-3′ |
| Mouse Arg-1 | Forward: 5′-CATATCTGCCAAAGACATCGTG-3′ |
|  | Reverse: 5′-GACATCAAAGCTCAGGTGAATC-3′ |
| Mouse IL-10 | Forward: 5′-TGGCCCAGAAATCAAGGAGC-3′ |
|  | Reverse: 5′-CAGCAGACTCAATACACACT-3′ |
| Mouse TGF-β | Forward: 5′-CTCCCGTGGCTTCTAGTGC-3′ |
|  | Reverse: 5′-GCCTTAGTTTGGACAGGATCTG-3′ |
| Mouse iNOS | Forward: 5′-CAGAGGACCCAGAGACAAGC-3′ |
|  | Reverse: 5′-TGCTGAAACATTTCCTGTGC-3′ |
| Mouse SIRT3 | Forward: 5′-CTGACTTCGCTTTGGCAGAT-3′ |
|  | Reverse: 5′-GTCCACCAGCCTTTCCACAC-3′ |
| Human SIRT3 | Forward: 5′-GCCATTTTTGAACTCCCATTCT-3′ |
|  | Reverse: 5′-GGAGAAAGTAGTGAGTGACGTT-3′ |
| Mouse ETV4 | Forward: 5′-CACAGACTTCGCCTACGACTCA-3′ |
|  | Reverse: 5′-GCAGACATCATCTGGGAATGGTC-3′ |
| Mouse U6 | Forward: 5′-GCTTCGGCAGCACATATACTAAAAT-3′ |
|  | Reverse: 5′-CGCTTCACGAATTTGCGTGTCAT-3′ |
| Mouse GAPDH | Forward: 5′-AGGTCGGTGTGAACGGATTTG-3′ |
|  | Reverse: 5′-TGTAGACCATGTAGTTGAGGTCA-3′ |
| Human GAPDH | Forward: 5′-GAAGGTGAAGGTCGGAGTCA-3′ |
|  | Reverse: 5′-GAAGATGGTGATGGGATTTC-3′ |
